# Theoretical Characterization of the Step-by-Step Mechanism of Conversion of Leukotriene A_4_ to Leukotriene B_4_ Catalysed by the Enzyme Leukotriene A_4_ Hydrolase

**DOI:** 10.3390/ijms23063140

**Published:** 2022-03-15

**Authors:** Miquel Canyelles-Niño, Àngels González-Lafont, José M. Lluch

**Affiliations:** 1 Departament de Química, Universitat Autònoma de Barcelona, 08193 Bellaterra, Barcelona, Spain; miquel.canyelles@uab.cat (M.C.-N.); angels.gonzalez@uab.cat (À.G.-L.); 2 Biochemize SL, Carrer de Zamora, 45, 08005 Barcelona, Barcelona, Spain; 3 Institut de Biotecnologia i de Biomedicina (IBB), Universitat Autònoma de Barcelona, 08193 Bellaterra, Barcelona, Spain

**Keywords:** leukotriens, leukotriene A_4_ hydrolase, enzyme catalysis, QM/MM calculations, molecular dynamics simulations, proinflammatory lipid mediators

## Abstract

LTA_4_H is a bifunctional zinc metalloenzyme that converts leukotriene A_4_ (LTA_4_) into leukotriene B_4_ (LTB_4_), one of the most potent chemotactic agents involved in acute and chronic inflammatory diseases. In this reaction, LTA_4_H acts as an epoxide hydrolase with a unique and fascinating mechanism, which includes the stereoselective attachment of one water molecule to the carbon backbone of LTA_4_ several methylene units away from the epoxide moiety. By combining Molecular Dynamics simulations and Quantum Mechanics/Molecular Mechanics calculations, we obtained a very detailed molecular picture of the different consecutive steps of that mechanism. By means of a rather unusual 1,7-nucleophilic substitution through a clear S_N_1 mechanism, the epoxide opens and the triene moiety of the substrate twists in such a way that the bond C_6_-C_7_ adopts its *cis* (*Z*) configuration, thus exposing the *R* face of C_12_ to the addition of a water molecule hydrogen-bonded to ASP375. Thus, the two stereochemical features that are required for the bioactivity of LTB_4_ appear to be closely related. The noncovalent π-π stacking interactions between the triene moiety and two tyrosines (TYR267 and, especially, TYR378) that wrap the triene system along the whole reaction explain the preference for the *cis* configuration inside LTA_4_H.

## 1. Introduction

Nowadays, it is recognised that inflammatory-based human diseases represent the leading causes of present-day morbidity and mortality worldwide [1]. Infection of the host, tissue injury or surgical trauma trigger the release of proinflammatory lipid and protein mediators by cells in the site of challenge. These chemical mediators act as chemoattractants to recruit neutrophiles. This initial inflammatory response occurs to protect the host and produces the very well know signs of acute inflammation (redness, heat, swelling and pain) [2]. After this onset phase, the inflammatory reaction must be resolved to prevent the inflammation from spreading. The resolution phase is regulated by a number of specialised proresolving lipid mediators (produced by human cells called macrophages). If resolution does not work well, the acute inflammation evolves to chronic inflammation [3,4,5,6,7]. Chronic inflammatory diseases are the most significant cause of death in the world today [1]. 

**Figure 1:**
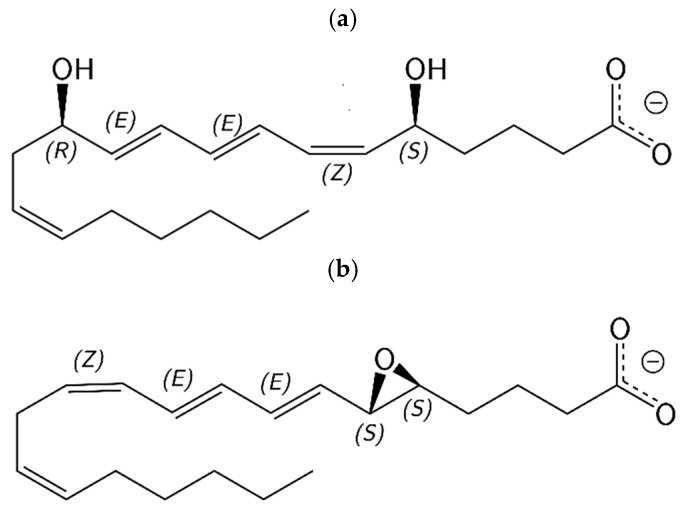
Structures corresponding to LTB_4_ (**a**) and LTA_4_ (**b**).

The lipid mediator Leukotriene B_4_ (LTB_4_, 5*S*,12*R*-dihydroxy-6*Z*,8*E*,10*E*,14*Z*-eicosatetraenoic acid) (Figure 1a) exerts one of the most potent chemotactic effects on polymorphonuclear leukocytes in the onset phase. In addition, excessive formation of LTB_4_ can be related to the maintenance of chronic inflammatory diseases [8]. The biosynthesis of LTB_4_ from arachidonic acid (AA) is the result of the action of several proteins. To start, the 5-lipoxygenase-activating protein (FLAP), an integral membrane protein, facilitates the transfer of the substrate AA to the enzyme 5-lipoxygenase (5-LOX) [9,10,11]. 5-LOX produces 5*S*-hydroperoxy-6*E*,8*Z*,11*Z*,14*Z*-eicosatetraenoic acid (5-HpETE) as a result of the hydroperoxidation of C5 of AA. Then, 5-LOX catalyses the formation of the highly labile epoxide intermediate Leukotriene A_4_ (LTA_4_, 5*S*,6*S*-epoxy-7*E*,9*E*,11*Z*,14*Z*-eicosate-traenoic acid) (Figure 1b) by dehydration of 5-HpETE. FLAP regulates the 5-LOX activity, increasing the efficiency of the formation of LTA_4_ from 5-HpETE [8,12,13,14]. Finally, after having been released from 5-LOX, LTA_4_ is hydrolased into LTB_4_ by the enzyme leukotriene A_4_ hydrolase (LTA_4_H) (EC 3.3.2.6) [8].

LTA_4_H is a monomeric bifunctional zinc metalloenzyme. It is detected in almost all mammalian cells, and it has both aminopeptidase activity and epoxide hydrolase activity. In this paper, we will focus on the second one. This epoxide hydrolase reaction is interesting because involves the stereoselective introduction of one water molecule to the carbon backbone (C_12_) of LTA_4_ in a position several methylene units away from the epoxy moiety (carbons C_5_ to C_6_) [15]. 

A high-resolution crystal structure of human LTA_4_H in the complex with the competitive inhibitor bestatin revealed that the protein includes three domains, an N-terminal domain, a Zn-containing catalytic domain and an α-helical C-terminal domain. The active site is placed in a deep cleft in between the domains, where the Zn^2+^ is coordinated to HIS295, HIS299 and one carboxylic oxygen of GLU318 [16,17]. Because of the LTA_4_ instability (half-life of about 10 s at neutral pH) high-resolution crystal structures of LTA_4_H complexed to LTA_4_ were not determined until recently by Haeggström and coworkers [18]. They described six different structures of human LTA_4_H from five distinct crystal forms, finding two conformational states of the enzyme and several conformations of the substrate LTA_4_, and showing that LTA_4_H undergoes domain movements. From this structural information and previous site-directed mutagenesis experiments [15,19,20], Haeggström and coworkers [18] propose a mechanism for the epoxide hydrolase reaction. In short, the C_1_ carboxylate group of LTA_4_ anchors to ARG563, while Zn^2+^ and TYR383 are coordinated to the epoxide oxygen. A catalytic water molecule (WAT1), polarised by Zn^2+^ and GLU271, transfers a proton to produce an S_N_1 acid-induced opening of the epoxide ring. The positive charge of the resulting carbonium ion is delocalised over the triene system, with the C_6_-C_7_ bond in a pro-*cis* configuration stabilised by the L-shape of the tight pocket. Finally, a second water molecule, activated by ASP375, adds to C_12_ to generate an *R*-hydroxyl group. It has to be underlined that the 12*R*-hydroxyl group and the 6*Z*,8*E*,10*E* configuration of the conjugated triene system are required for the bioactivity of LTB_4_ [15]. It seems that ASP375 is needed for the generation of the former [15], whereas TYR378 could be involved in the formation of the right configuration of the triene moiety [21,22].

To our knowledge, only a theoretical study of the epoxide hydrolase reaction exists so far [23]. However, this work was just restricted to the step corresponding to the epoxy ring opening, using the self-consistent-charge density-functional tight-binding (SCC-DFTB) theory [24,25,26] for the Quantum Mechanics calculations and the CHARMM force field for the molecular mechanics part [27]. Thus, considering only the first reaction step and using a low level of calculation, it provided a rather incomplete view of the whole reaction mechanism to obtain LTB_4_.

In this paper, we combine Molecular Dynamics (MD) simulations and Quantum Mechanics/Molecular Mechanics (QM/MM) calculations, using the B3LYP hybrid functional to describe the QM part, to disclose the molecular details of the complete step-by-step mechanism of conversion of leukotriene A_4_ to leukotriene B_4_ catalysed by the enzyme leukotriene A_4_ hydrolase. In particular, we analyse the key factors that drive the 12*R* and the 6*Z*,8*E*,10*E* stereochemistries of the hydroxyl group and the triene moiety, respectively, that are essential for the bioactivity of LTB_4_.

## 2. Results and Discussion

### 2.1. QM/MM Structural Analysis

**Figure 2:**
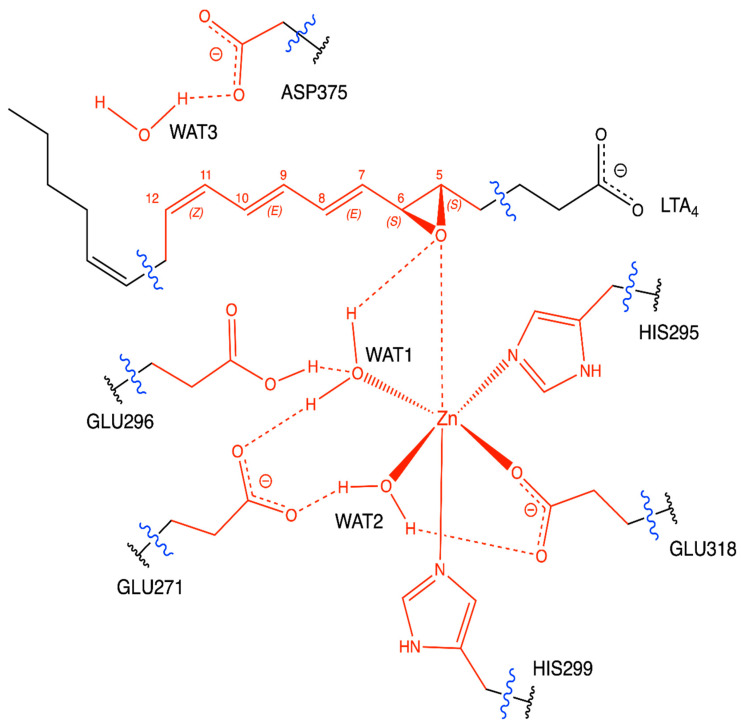
QM/MM partition. QM atoms are depicted in red. The boundary between the QM and MM regions is indicated by blue wavy lines.

**Figure 3:**
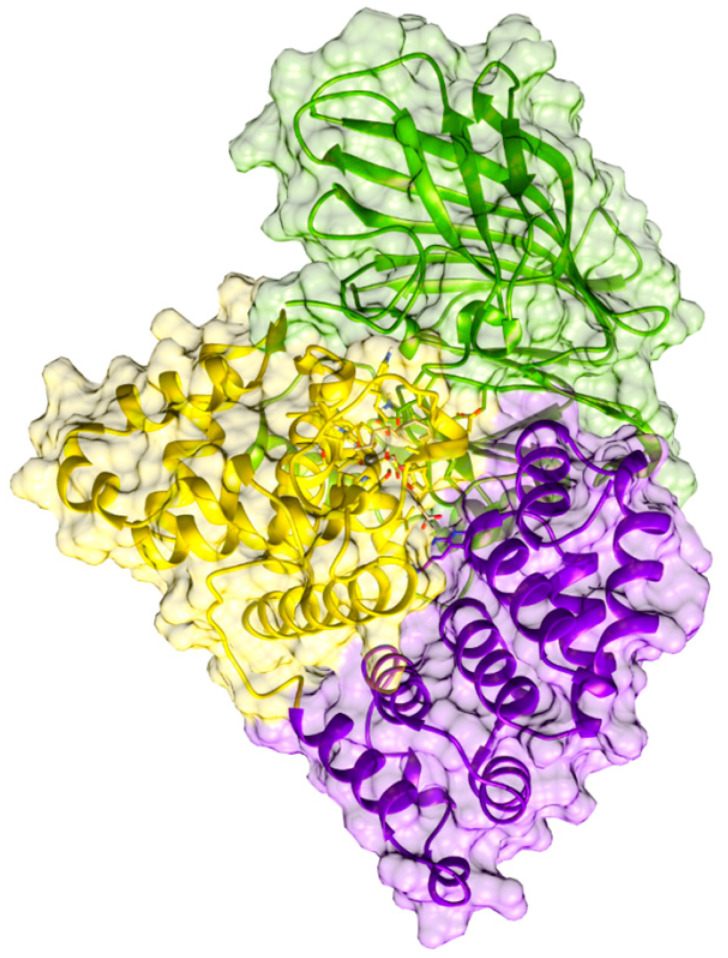
Complete view of the QM/MM-optimised LTA_4_:LTA_4_H Michaelis complex (the solvation waters are not shown for the sake of clarity). LTA_4_H has three domains that are pictured here in different colours: The N-terminal domain (in green), the catalytic domain (in yellow) and the C-terminal domain (in violet). The substrate LTA_4_ is represented using a stick model.

**Figure 4:**
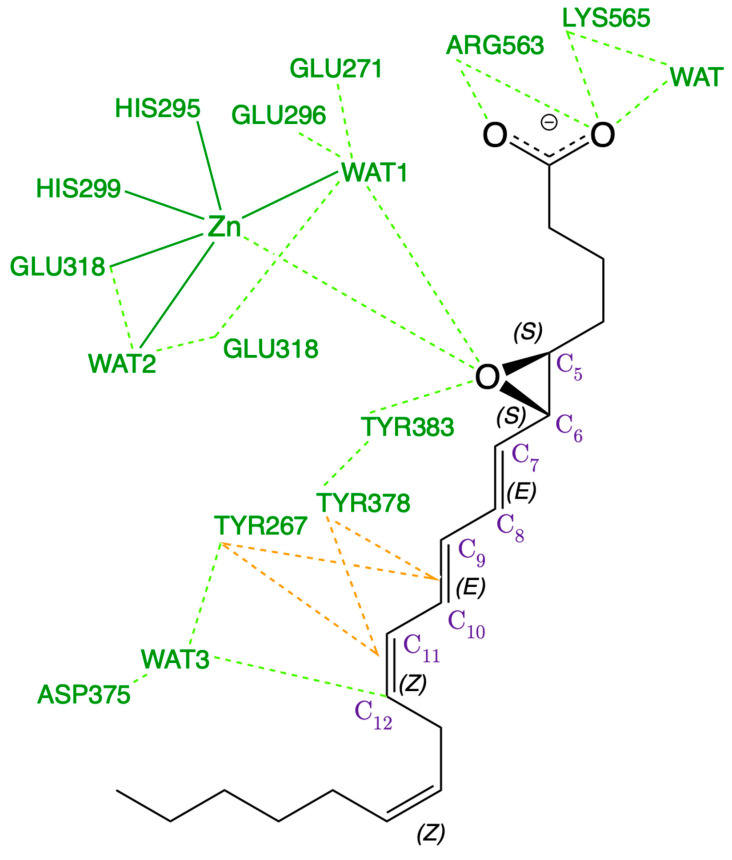
Scheme of the main noncovalent interactions (in dashed lines) between the substrate LTA_4_ (in black) and the enzyme LTA_4_H (in green) in the optimised Michaelis complex and the Zn environment. The dashed orange lines indicate π-π stacking interactions.

According to the crystallographic structures [18], the catalytic water molecule WAT1 is hydrogen-bonded to two Glutamate residues, GLU271 and GLU296. However, the positions of the corresponding hydrogen atoms are not a priori clear. That is, which of these Glutamate residues, GLU271 or GLU296, is the protonated one is not known. Thus, as explained below, two versions of the protonation state of the LTA_4_:LTA_4_H Michaelis complex have been prepared starting from (GLU271, protonated GLU296) or (protonated GLU271, GLU296), leaving all the other protonation states untouched. These two initial structures have been QM/MM-optimised, and the same minimum energy structure was obtained in both cases, showing that the protonated residue is GLU296, as pictured in Figure 2, where the QM/MM partition used is also shown. Thus, WAT1 coordinates to GLU271 through a donor water hydrogen bond, while it connects to GLU296 through an acceptor hydrogen bond. The remaining water hydrogen atom is the one interacting with the oxygen atom of the epoxide. A complete view of this QM/MM-optimised LTA_4_:LTA_4_H Michaelis complex is pictured in Figure 3. A scheme of the main noncovalent interactions between the substrate LTA_4_ and the enzyme LTA_4_H in this optimised Michaelis complex is shown in Figure 4.

LTA_4_ fits into the L-shaped hydrophobic cavity of the LTA_4_H. The substrate docks to the protein through the interaction between the polar head of the LTA_4_ and ARG563 and LYS565 from the protein, which are located in the depth of the cavity. Thus, the substrate is placed in a head-first conformation. Moreover, this fitting conformation lets the epoxide oxygen move near the Zn environment, while it places C_12_ near ASP375 (with WAT3 between them). The epoxide is not only stabilised by being in the second coordination sphere of the Zn atom, but also thanks to two hydrogen bonds from TYR383 and WAT1, the catalytic water which is also coordinated to Zn. With this conformation, TYR267 and TYR378 are the two residues closest to the triene moiety of the substrate. The main interactions between the substrate LTA_4_ and the enzyme LTA_4_H in this optimised Michaelis complex are schematised in Figure 4. The role of all these residues is to limit the available space and give a shape to the cavity. TYR267 and TYR378 are key residues of the selectivity of the reaction too (see below).

**Figure 5:**
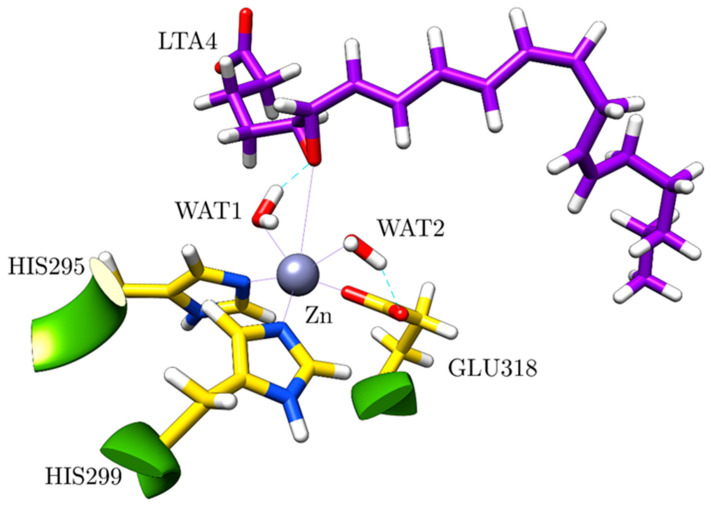
Zn environment in the optimised structure.

The Zn ion is the central atom of the catalytic domain of the protein. In good agreement with the crystallographic structures [18], in the optimised structure it is pentacoordinated to two HISs (HIS295, HIS299), GLU318, WAT1 and WAT2 with an octahedral geometry (distance RMSD compared to the ideal octahedron: 0.490 Å) [28], so a vacancy for the coordination is available (Figure 5). The free O from GLU318 is linked to WAT2 through a hydrogen bond. Moreover, there is a second coordination sphere, which initially contains the epoxide of LTA_4_, GLU271 (hydrogen-bonded to WAT1 and WAT2) and GLU296 (linked to WAT1 through a hydrogen bond).

### 2.2. Molecular Dynamics Simulation

In the above section we have analysed the structure of the optimised LTA_4_:LTA_4_H Michaelis complex and we have identified some key points for the reaction mechanism. However, we might wonder if these features correspond just to this particular minimum energy structure, or if they hold throughout the dynamic fluctuations of the enzyme system. To unravel this point, we analysed 100 ns extracted from a Molecular Dynamics simulation of that Michaelis complex carried out following the protocol explained below. The QM/MM-optimised structure described in Section 2.1. was taken as starting point for the MD simulation.

**Figure 6:**
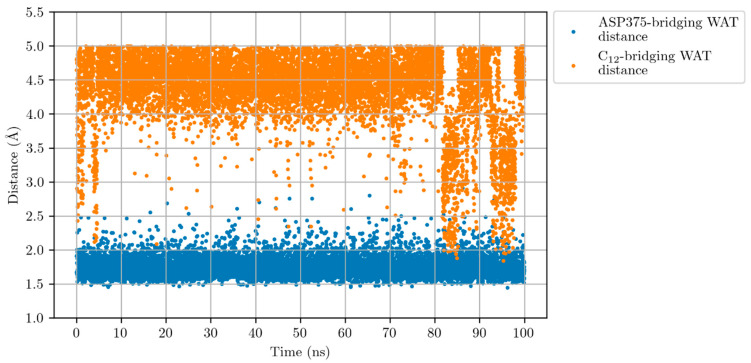
Distances ASP375-WAT (blue points) and C_12_-WAT (orange points) along the Molecular Dynamics simulation for the LTA_4_:LTA_4_H Michaelis complex. WAT stands for a water molecule placed between ASP375 and C_12_.

Firstly, we selected the snapshots along the Molecular Dynamics simulation where there is a water molecule with a distance ASP375-WAT up to 3 Å and a distance C_12_-WAT smaller than 5 Å at once. In Figure 6, we illustrate where the water molecule appears in those selected snapshots as a function of the distances ASP375-WAT and C_12_-WAT. This water molecule is the one that appears between ASP375 and C_12_. Along the simulation there is exchange between the water molecules, in such a way that the water molecule that is represented in this figure is not always the same. In the optimised QM/MM structure, this is WAT3. Almost all the snapshots (96% of the generated snapshots) include a bridging water molecule closer than 3 Å and 5 Å to ASP375 and C_12,_ respectively. Even as many as 58% of the generated snapshots involve a bridging water molecule closer than 3 Å and 4.5 Å to ASP375 and C_12,_ respectively. It is clear that in most configurations a water molecule, activated by ASP375, appears to be ready to attack C_12_, thus forming a hydroxyl group.

**Figure 7:**
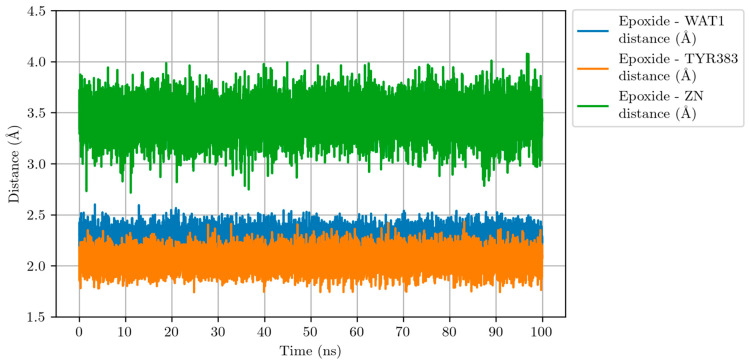
Distances epoxide-WAT1 (blue line), epoxide-TYR383 (orange line) and epoxide-Zn (green line) along the Molecular Dynamics simulation for the LTA_4_:LTA_4_H Michaelis complex.

On the other hand, the evolution of distances shown in Figure 7 confirms that the epoxide of LTA_4_ maintains two hydrogen bonds with TYR383 and WAT1, and that it remains in the second coordination sphere of Zn ion along the Molecular Dynamics simulation.

**Figure 8:**
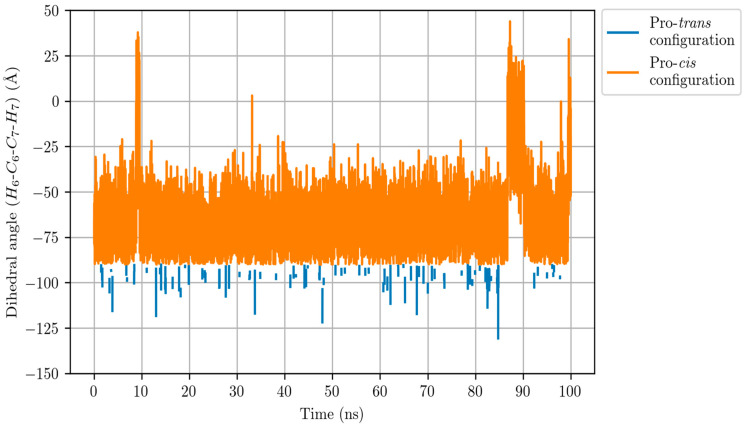
Dihedral angle H_6_-C_6_-C_7_-H_7_ along the Molecular Dynamics simulation for the LTA_4_:LTA_4_H Michaelis complex. The pro-*cis* configuration (orange line) corresponds to |dihedral angle|≤90°, whereas |dihedral angle|≥90° indicates a pro-*trans* configuration (blue line).

Finally, we can see in Figure 8 that the C_6_-C_7_ bond of LTA_4_ (the single bond next to the epoxide) keeps a rather pro-*cis* configuration along the Molecular Dynamics simulation. A total of 91% of the snapshots correspond to a pro-*cis* configuration |dihedral angle H6−C6−C7−H7|≤90°, whereas only 9% are associated with a pro-*trans* configuration |dihedral angle H6−C6−C7−H7|≥90°. The evolution of this dihedral angle along the complete enzyme reaction is a key point for the formation of LTB_4_, as will be explained below.

### 2.3. QM/MM Reaction Mechanism Calculations

**Figure 9:**
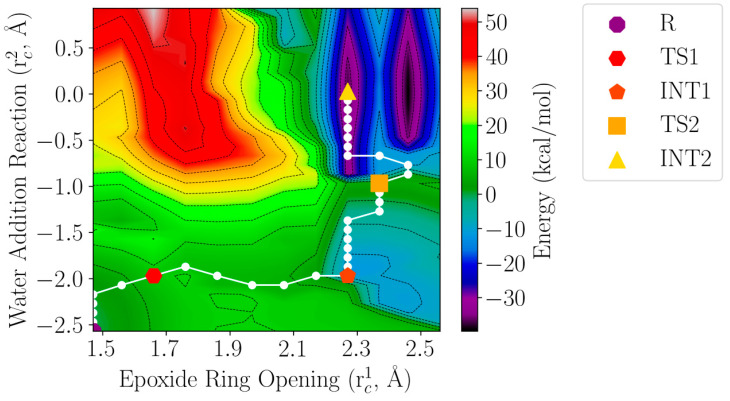
Two-dimensional potential energy surface as a function of the epoxide ring opening and water addition reaction coordinates defined in the text. Isoenergetic lines correspond to intervals of 5 kcal/mol.

The conversion of LTA_4_ to LTB_4_ is unique because the water molecule is proposed [15,17] to be introduced stereoespecifically at a site (C_12_) six methylene units away from the epoxide moiety (C_5_ to C_6_), in what would be a 1,7-nucleophilic substitution. Although it is believed [17] to proceed by means of an S_N_1 mechanism, an S_N_2 mechanism should also be considered as possible. To unravel this point, a two-dimensional potential energy surface was built (see Figure 9) using a reaction coordinate $r_{c}^{1}$ for the epoxide ring opening ($r_{c}^{1}$ = d(C_6_(LTA_4_) − O_epox_(LTA_4_)), that is, the epoxide breaking bond length, and a reaction coordinate $r_{c}^{2}$ for the water addition to C_12_ ($r_{c}^{2}$ = (d(O(WAT3) − H(WAT3)) − d(O(WAT3) − C_12_(LTA_4_))), that is, the difference between the water breaking bond length and the forming O–C bond length. The rest of the geometrical parameters in the active region have been fully optimised. R stands for the optimised structure (Figure 3, Figure 4 and Figure 5). Using the Dijsktra algorithm [29] we have also defined in Figure 6 the minimum energy reaction path (white points) on that surface and the minimum (R, INT1 and INT2) and maximum energy points along it (TS1 and TS2).

As shown in Figure 9, the reaction takes place in two well-separated steps. Firstly, the C_6_ − O_epox_ bond progressively breaks, thus opening the epoxide ring. In the intermediate INT1, the epoxide is already open (the distance C_6_ − Oepox is 2.3 Å), but the value of the reaction coordinate $r_{c}^{2}$ has not significantly changed yet. From here on, in the second step the distance C6 − Oepox remains quite invariant, while a clear evolution of the reaction coordinate $r_{c}^{2}$ occurs, indicating the water (WAT3) addition to C_12_ and the breakage of an O-H bond in this water molecule and finally reaching the intermediate INT2. Thus, the 1,7-nucleophilic substitution clearly takes place through an S_N_1 mechanism. As a matter of fact, it is an extreme case of S_N_1 reaction, because the minimum energy reaction paths corresponding to each step appear to be roughly orthogonal (each one parallel to one coordinate axis) in the two-dimensional potential energy surface shown in Figure 9. Any deviation of the mechanism towards the region near the diagonal of that energy surface (that is, any approach to the S_N_2 mechanism) implies the penetration in high-energy regions, which turns out to be forbidden. An NBO charge analysis in INT1 gives a charge of +0.71 a.u. delocalised over the triene system (from C_6_ to C_12_) of the substrate, which confirms the existence of the carbocation required in the intermediate of an S_N_1 mechanism.

**Figure 10:**
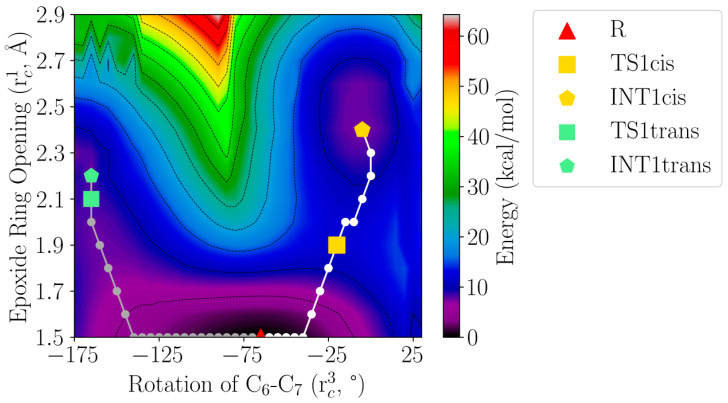
Two-dimensional potential energy surface as a function of the rotation of the C_6_-C_7_ bond and the epoxide ring-opening reaction coordinates defined in the text. Isoenergetic lines correspond to intervals of 5 kcal/mol.

To our surprise, a structural analysis of the intermediate INT2 shows that the conjugated triene moiety has a 6*E*,8*E*,10*E* configuration that would not lead to LTB_4_ but to its stereoisomer 12*S*-6-*trans*-LTB_4_ (5*S*,12*S*-dihydroxy-6*E*,8*E*,10*E*,14*Z*-eicosatetraenoic acid). Following the evolution of the structures along the S_N_1 mechanism shown in Figure 9, we found that an unexpected rotation of the C_6_-C_7_ bond from the initial rather pro-*cis* configuration in LTA_4_ to a pro-*trans* configuration in INT1 occurs, which leads to the 6*E* inadequate configuration of C_6_-C_7_ in INT2. To analyse this fact, we built a two-dimensional potential energy surface (see Figure 10) as a function of the rotation of the C_6_-C_7_ bond ($r_{c}^{3}$ = dihedral angle H_6_-C_6_-C_7_-H_7_) and the epoxide ring opening ($r_{c}^{1}$ = d(C_6_(LTA_4_) − O_epox_(LTA_4_)). Starting from the reactant R, the Dijsktra algorithm [29] localises two minimum energy reaction paths (grey and white points) on the surface shown in Figure 10, and the minimum (R, INT1trans and INT1cis) and maximum (TS1trans and TS1cis) energy points along them. One path involves the pro-*trans* (grey points) configuration (as a matter of fact, it corresponds to the first step of the S_N_1 mechanism shown in Figure 9). The other one maintains a pro-*cis* configuration (white points) to reach an intermediate INT1cis with the adequate pro-*cis* structure. The two paths are competitive, with the pro-*trans* being the one that implies the lowest potential energy barrier. This explains why only the *trans* configuration was obtained in the two-dimensional potential energy surface shown in Figure 9, where the rotation of the C_6_-C_7_ bond was not included to define the reaction coordinate. This result also agrees with our B3LYP/6-31G(d) calculation that shows that in the gas phase, 12*S*-6-*trans*-LTB_4_ is 0.3 kcal/mol more stable than LTB_4_ in terms of potential energy. However, the experimental result is that LTA_4_H converts LTA_4_ into LTB_4_, that is, with a 6*Z*,8*E*,10*E* configuration for the conjugated triene moiety.

**Figure 11:**
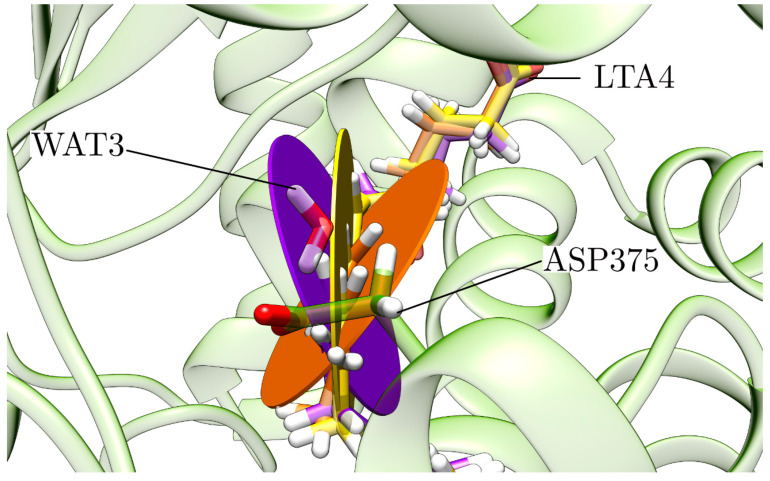
Position of WAT3 with respect to the plane generated by the triene moiety in the reactant LTA_4_ (pro-*cis*, yellow disk), in INT1trans (pro-*trans*, brown disk) and in INT1cis (pro-*cis*, violet disk).

An especially interesting point is that the configuration of the C_6_-C_7_ bond in INT1 determines the stereochemistry of the ingoing water into C_12_. In Figure 11, we have displayed the position of WAT3 with respect to the plane generated by the triene moiety (from C_6_ to C_12_) in the reactant LTA_4_ (pro-*cis*, yellow disk) in INT1trans (pro-*trans*, brown disk) and in INT1cis (pro-*cis*, violet disk). It can be clearly seen that the face of attack of WAT3 in the pro-*trans* configuration is just the opposite than in the case of the pro-*cis* configuration in INT1, leading to the 12*S*-hydroxyl or the 12*R*-hydroxyl configurations, respectively. Thus, the two main stereochemical features that are required for the bioactivity of LTB_4_ appear to be closely linked.

**Figure 12:**
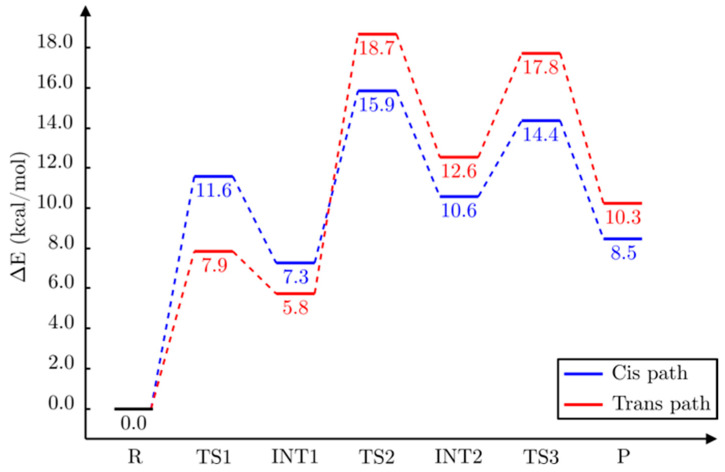
Diagram of potential energies of the stationary structures of the complete mechanism of conversion of LTA_4_ to LTB_4_ (pro-*cis* reaction path, in blue) catalysed by LTA_4_H and of the competitive pro-*trans* reaction path (in red).

At this point, it is clear that a complete and comparative study of the pro-*cis* and the pro-*trans* reaction paths is needed to understand the mechanism of this enzyme reaction. To this aim, firstly we fully optimised the structures INT1cis, INT1trans, TS1cis and TS1trans, which appear in Figure 10, in order to locate the corresponding stationary structures (minima and transition-state structures) in the complete potential hypersurface of the reaction. Then, for each reaction path, we followed the reaction coordinates d(O(WAT3)-H(WAT3))-d(O(WAT3)-C_12_(LTA_4_)) and d(O(WAT1)-H(WAT1))-d(H(WAT1)-O_epox_) to describe, respectively, the water addition (WAT3) to C_12_ and the final protonation of the oxygen atom bonded to C_6_ (the former oxygen atom of the epoxide) by WAT1 and to locate the corresponding stationary structures. The relative potential energies for all the stationary points we localised are shown in Figure 12.

**Figure 13:**
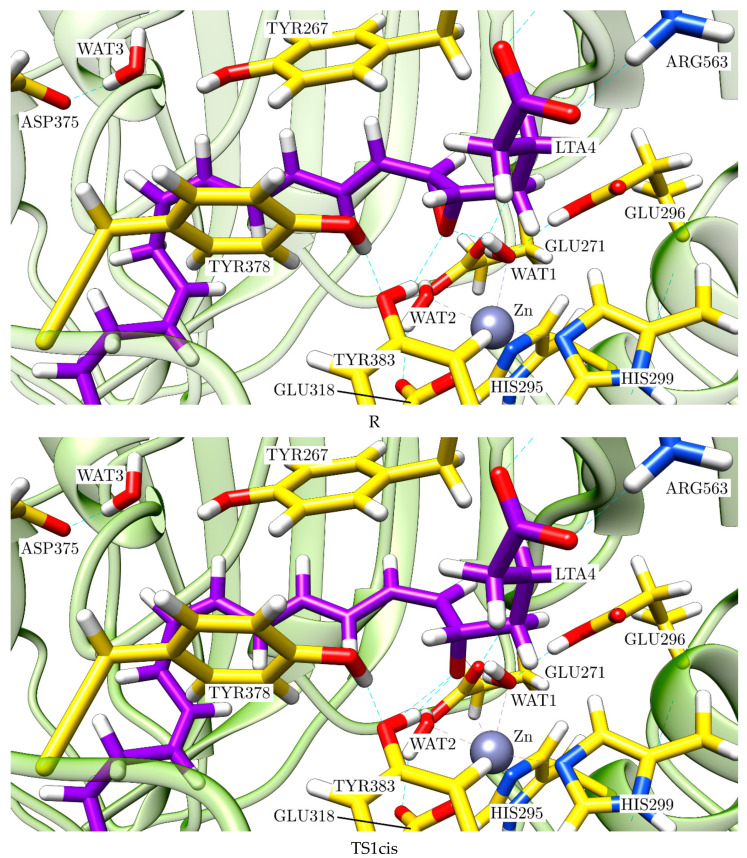

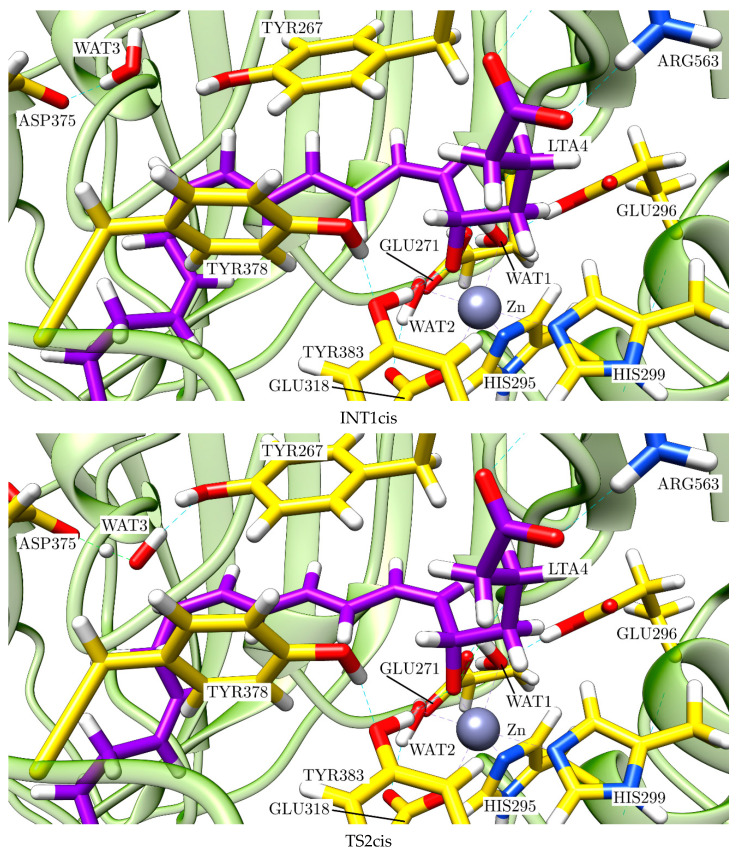

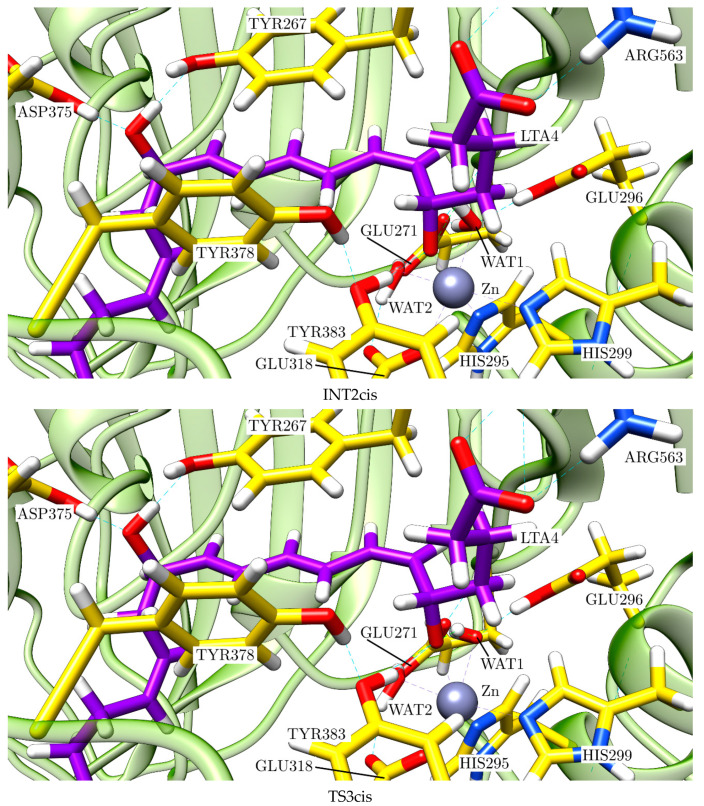

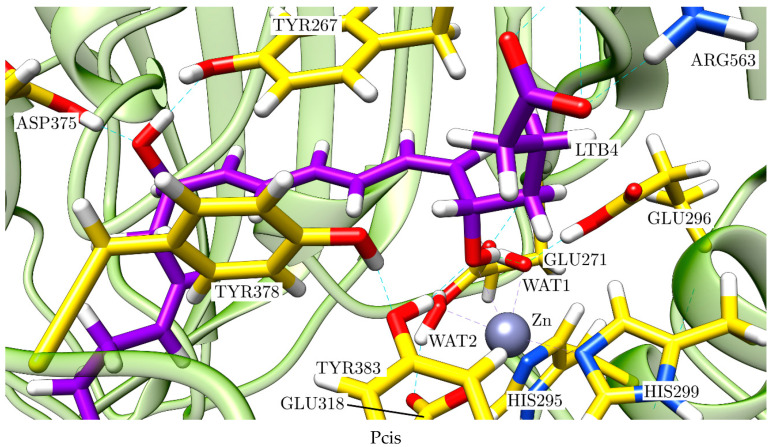
Stationary structures of the complete mechanism of conversion of LTA_4_ to LTB_4_ (pro-*cis* reaction path) catalysed by LTA_4_H: R, TS1cis, INT1cis, TS2cis, INT2cis, TS3cis and Pcis.

We focused firstly on the pro-*cis* reaction path. In Figure 13 we show a succession of pictures of the stationary structures along this path, intending to capture LTA_4_H in action. For the sake of clarity, in Figures S1–S3 we display representations of those stationary structures focused on the region where the corresponding step occurs in each case.

The evolution of the main geometrical parameters along the pro-*cis* reaction path leading to LTB_4_ is indicated in Table 1. As indicated above, the first step consists of the epoxide opening. The formation of a nascent negative charge in the oxygen of the epoxide is stabilised by the interaction of this oxygen with the Zn atom, the hydroxyl group of TYR383 and the hydrogen bond interaction with WAT2 and WAT1, although WAT1 moves away from INT1cis. Conversely, the Zn-O_epox_ bond is significantly shortened from 3.39 Å in R to 1.99 Å at INT1cis, in such a way that epoxide completes the coordination sphere of Zn by occupying its free vacancy (see Figure 5). The epoxide is fully opened at INT1cis, while O_epox_-C_5_ is stronger at INT1cis than in R. This step implies a potential energy barrier of 11.6 kcal/mol. As explained above, at INT1cis no significant changes have occurred with respect to WAT3 and ASP375 yet, thus indicating an S_N_1 mechanism. Very interestingly, the dihedral angle H_6_-C_6_-C_7_-H_7_ goes from −65.6° in R (a value close to an intermediate situation of the bond C_6_-C_7_ between pro-*cis* and pro-*trans*) to −15.9° at TS1cis and −7.1° at INT1cis, a clearly *cis* stereochemistry. As a result of the twist that the triene moiety must perform to adopt this *cis* stereochemistry, WAT3 becomes ready to attack the *R* face of C_12_ (by the side opposite to the Zn atom, see Figure S2) at INT1cis. Then, WAT3 adds to C_12_ (the distance O(WAT3)-C_12_ evolves from 3.69 Å at INT1cis to 1.53 Å at INT2cis), while one of its hydrogen atoms is fully transferred to ASP375 through the corresponding hydrogen bond. This second step involves a potential energy barrier of 15.9 kcal/mol. Finally, in the third step, the final transfer of a proton from WAT1 to the oxygen atom bonded to C_6_ (the former oxygen atom of the epoxide, O_epox_) takes place, while the distance Zn-O_epox_ clearly increases through a potential energy barrier of 14.4 kcal/mol, thus forming LTB_4_. Note that Haeggström and coworkers [18] have proposed that the catalytic water WAT1 transfers a proton to promote an S_N_1 acid-induced opening of the epoxide ring. However, we have found here that this proton donation is not the first step of the reaction, but the last one. That is, when the epoxide ring opens, the nascent negative charge in its oxygen atom is quite stabilised through the interaction with atoms surrounding it, in such a way that a previous proton transfer from WAT1 is not needed.

**Figure 14:**
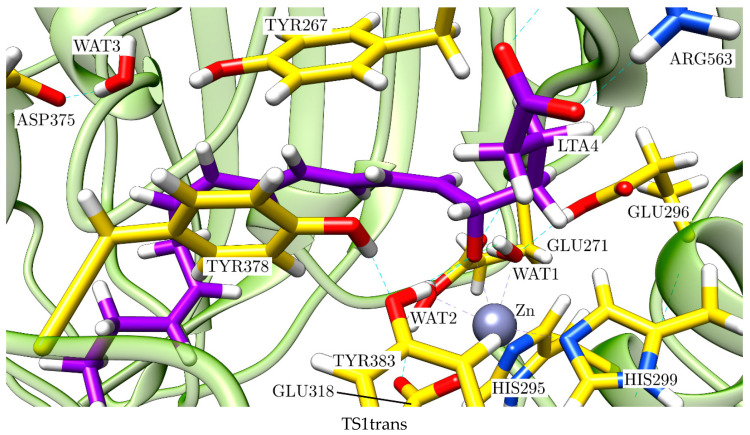

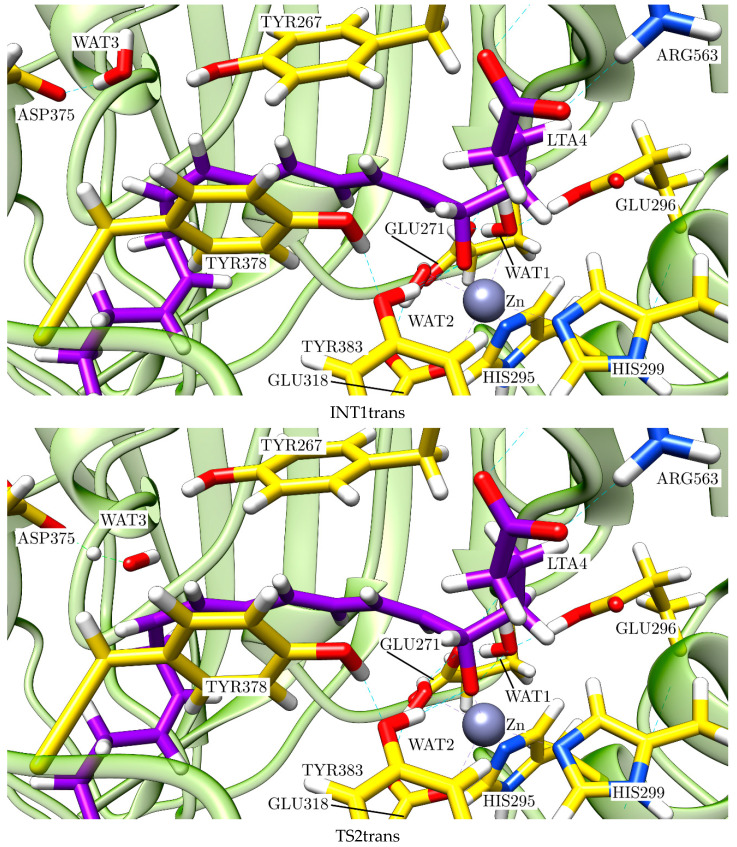

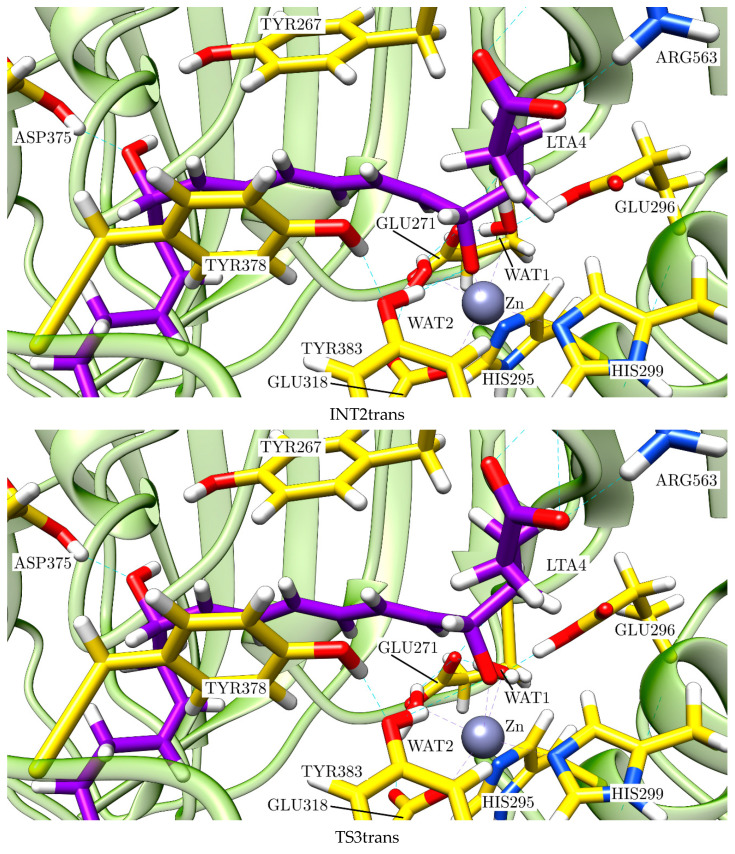

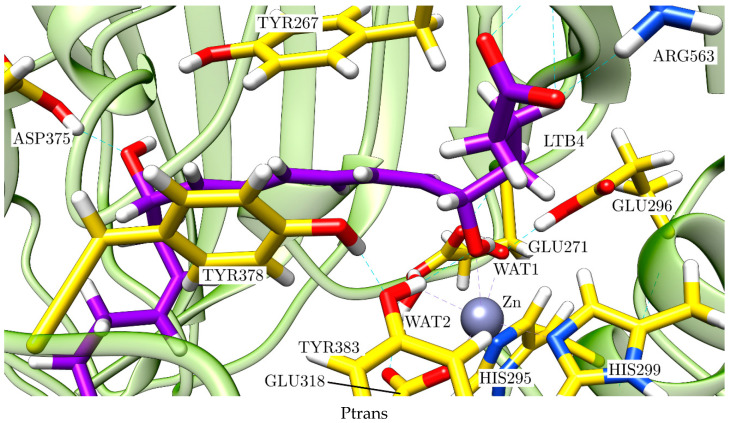
Stationary structures of the complete mechanism of the pro-*trans* reaction path catalysed by LTA_4_H: TS1trans, INT1trans, TS2trans, INT2trans, TS3trans and Ptrans.

Let us describe the pro-*trans* reaction path now. In Figure 14, we show a succession of pictures of the stationary structures along this path. For the sake of clarity, in Figures S4–S6, we display representations of those stationary structures focused on the region where the corresponding step occurs in each case.

The evolution of the main geometrical parameters along the pro-*trans* reaction path is indicated in Table 2. Analogously to the case of the pro-*cis* reaction path, in the first step of the pro-*trans* reaction path, the oxygen of the epoxide is stabilised by the interaction with the Zn atom, the hydroxyl group of TYR383 and the hydrogen bond interaction with WAT2 and WAT1, although WAT1 also moves away at INT1trans. Conversely, the Zn-O_epox_ bond is significantly shortened from 3.39 Å in R to 1.98 Å at INT1trans. The epoxide is fully opened at INT1trans of this S_N_1 mechanism. This step implies a potential energy barrier of only 7.9 kcal/mol. The key stereochemical features of the complete reaction are determined in this first step, where the fate of the process is decided. Thus, the dihedral angle H_6_-C_6_-C_7_-H_7_ is 196.9° at TS1trans and 183.7° at INT1trans, a clearly *trans* stereochemistry. This twist of the triene moiety takes place in the opposite direction to the twist that happens in the pro-*cis* reaction path. This way, WAT3 now becomes ready to attack the *S* face of C_12_ (by the same side as the Zn atom, see Figure S5) at INT1trans. Then, WAT3 adds to C_12_ (the distance O(WAT3)-C_12_ evolves from 3.66 Å at INT1trans to 1.51 Å at INT2trans), while one of its hydrogen atoms is fully transferred to ASP375 through the corresponding hydrogen bond. This second step involves a potential energy barrier of 18.7 kcal/mol. Finally, in the third step, the final transfer of a proton from WAT1 to the oxygen atom bonded to C_6_ takes place, while the distance Zn-O_epox_ clearly increases through a potential energy barrier of 17.8 kcal/mol, thus forming 12*S*-6-*trans*-LTB_4_, the stereoisomer of LTB_4_.

**Figure 15:**
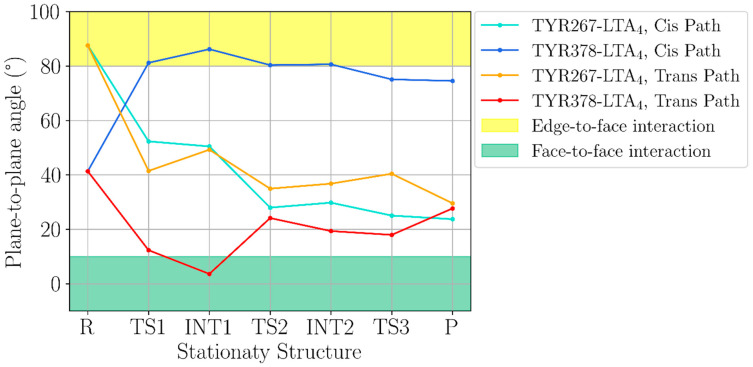
Angles between the plane of the triene moiety of the substrate and the planes of the aromatic rings of TYR267 and TYR378 for the different stationary structures located along the pro-*cis* and the pro-*trans* reaction paths. The closest plane to the whole number of C and H atoms of the triene system was taken as the plane of this triene moiety.

A final point to discuss is why LTA_4_H converts LTA_4_ into LTB_4_ instead of the stereoisomer 12*S*-6-*trans*-LTB_4_, which is more stable in the gas phase. In other words, why the pro-*cis* reaction path is the dominant one instead of the pro-*trans* reaction path. As seen in Figure 12, as for the epoxide opening the *trans* path is more favourable than the *cis* path. However, from here on, the *cis* energy profile appears clearly below the *trans* energy profile. In particular, taking into account the complete mechanism, the higher energy transition state structure of the *trans* path (18.7 kcal/mol) and the *trans* final product (10.3 kcal/mol) are significantly above the corresponding values of the *cis* path (15.9 kcal/mol and 8.5 kcal/mol, respectively), what means that the inside of the LTA_4_H formation of LTB_4_ is more favourable than the formation of 12*S*-6-*trans*-LTB_4_ both kinetically and thermodynamically. This energy crossing can be explained by observing some of the most important noncovalent interactions that take place between the substrate and the enzyme. TYR267 and TYR378 are the two residues closest to the triene system, which is practically wrapped by these two tyrosines along the reaction paths (see the sequence of structures shown in Figure 13 and Figure 14). The key point here is that these two tyrosines and the triene moiety can interact among them through noncovalent π-π stacking interactions that are different depending on the reaction path (see Figure 15 for the angles between the respective planes). The angle of the π-π interaction between TYR267 and TYR378 is kept quite invariant (between 50° and 60°) throughout the complete reaction for both reaction paths, and it has not been depicted. It has to be recalled here that the closer the planes to the face-to-face orientation, the more stabilising the interaction. Thus, TYR267 slightly favours the pro-*trans* reaction path up to INT1 (the first step), but the pro-*cis* reaction path in the second and third steps. Much more relevant is the role of TY378. As explained above, during the epoxide opening the triene moiety twists in two opposite directions depending on which reaction path it takes, pro-*cis* or pro-*trans*. As a result of this twist, the π-π stacking interaction with TY378 becomes clearly face-to-face (pro-*trans*) or clearly edge-to-face (pro-*cis*). At INT1trans (see Figure 11) the planes of the triene moiety and TY378 are roughly parallel. This way, TYR378 favours the formation of a broken epoxide with a *trans* C_6_-C_7_ bond. However, along the second and third steps, edge-to-face interaction is kept for the pro-*cis* reaction path, while the face-to-face interaction for the pro-*trans* reaction path becomes significantly broken, thus destabilising the formation of 12*S*-6-*trans*-LTB_4_ and leading to LTB_4_ as a product of the reaction catalysed by LTA_4_H. Indeed, those two tyrosines do not exist in the gas phase, where the 12*S*-6-*trans*-LTB_4_ is more stable than LTB_4_. Our theoretical result agrees with experimental data [21,22] showing that mutations of TY378 lead not only to LTB_4_ but also to products with a *trans* stereochemistry in the C_6_-C_7_ bond.

## 3. Materials and Methods

### 3.1. Protein Setup

Numerous crystallographic structures [18] have been reported for human LTA_4_H, some of them complexing a wide variety of inhibitors and a few of them containing the LTA_4_ substrate but including one or more mutations. Here, a single-mutated version of human LTA_4_H (ASP375ASN) complexed with LTA_4_ was used (PDB code: 5NI6) [18]. The mutation was reverted to the WT enzyme. The protein was protonated at pH 7.0 using PropKa3.0 [30] through a web interface (www.playmolecule.org, accessed on 1 July 2019). Two versions of the protonation state were prepared: (GLU271, protonated GLU296) and (protonated GLU271, GLU296), leaving all the other protonation states untouched. A box of TIP3P [31] water molecules was built around the protein for solvation. The box was built up considering 10 Å from the outermost protein’s atom in each direction of the space.

### 3.2. QM/MM Calculations

The solvation model was cropped, so only the water molecules inside a 17 Å radius sphere around whatever atom of the substrate were kept for the QM/MM calculations. The modular software package ChemShell [32,33] was used as the interface between the QM and the MM calculations. TURBOMOLE [34] was used for the density functional theory (DFT) calculations, while AMBER [35] force fields were employed for the MM calculations using the DL_POLY [36] module in ChemShell. AMBER’s ff14SB [37] force field was used for generating the protein MM parameters. GAFF2 [38] force field was used for parameterising the substrate (LTA_4_), whose RESP charges [39] were calculated using Gaussian09 [40]. An electrostatic embedding scheme [41] was employed in order to treat the QM mm interactions. Additionally, a link atom scheme was used to treat the QM/MM boundary by using the charge shift model [42]. No cut-offs were introduced for the nonbonding QM/MM and MM interactions [43].

The active region of the system was built by selecting all atoms within a radius of 15 Å around the oxygen atom of the epoxide group of the substrate. These atoms were allowed to move freely (almost 3000 atoms), while the nonactive ones were frozen (almost 8000 atoms). For the QM part of the system (see Figure 2), 86 atoms were selected involving the Zn and its immediate environment (HIS295, HIS299, GLU318, WAT1, WAT2), GLU271 and GLU296, from C_4_ to C_13_ of the substrate (thus including the epoxide and the triene group and ASP375 and its nearest water molecule (WAT3). From this selection, eight link atoms were added: six between the corresponding bonds C(MM)-C(QM) atoms of the six residues and two bonded to the aliphatic carbon atoms of the lipid substrate (placed between C_3_-C_4_ and C_13_-C_14_). Moreover, a microiterative scheme [44] was applied for the energy optimisation to minima. All residues containing at least one atom in the QM region were added to the ‘micro’ region of the microiterative scheme, while all other atoms were kept in the ‘macro’ region.

The QM/MM energy optimisations to minima and scan calculations were performed using the HDLCopt (hybrid delocalised internal coordinate) coordinates scheme [45] and the limited-memory Broyden–Fletcher–Goldfard–Shanno (L-BFGS) algorithm [46,47]. For optimisations to minima, tolerance was set to 0.0045 Bohr, while for potential energy surface (PES) calculations it was set to 0.01 Bohr. PES calculations have at least one angle, bond or bond distance differences fixed in order to carry out the exploration. The transition-state searches were carried out employing the HDLC coordinates scheme and the partitioned rational function optimiser (P-RFO) [48,49] combined with the L-BFGS algorithm. P-RFO and the L-BFGS algorithm were used as implemented in the HDLCopt module and the DL_FIND geometry optimisation library [50] of ChemShell, respectively. A set of core atoms including only the 6 atoms directly implicated in the reaction was defined in order to ease the calculation of the Hessian along the optimisation. Frequencies of the optimised structures were calculated using the force module from ChemShell for the whole QM region. The nature of the stationary structures was confirmed by means of the analysis of the number of imaginary frequencies.

We used the Dijsktra algorithm [29] to obtain the minimum energy reaction path on the two-dimensional potential energy surfaces.

The QM region was described by the B3LYP hybrid functional [51]. The 6-31G(d) Pople basis set [52] was employed for the C, H, O and N atoms, while the Stuttgart RLC ECP basis set [53] was used for the Zn atom.

### 3.3. Molecular Dynamics Simulation

The crystallographic structure optimised at the QM/MM level and with the proper protonation (deprotonated GLU271, protonated GLU296) was chosen as the initial structure for the MD simulation. Protein parameters and charges were obtained from the ff14SB [37] force field from AMBER [35]. The substrate was parametrised using the parmchk2 module from AmberTools [54]. It was optimised at the B3LYP/6-31G(d) level, and then Merz–Kollman RESP charges [39] were calculated. Zn parameters were obtained following the Seminario Method [55] and the bonded model from AMBER using MCPB.py [56]. All residues within 3 Å around Zn were included in the model (HIS295, HIS299, GLU318, WAT1, WAT2). The model has not been optimised since the global structure corresponds to a QM/MM minimum, but frequencies were calculated as well as Merz–Kollman RESP charges [39] at the B3LYP/6-31G(d) level. MM parameters were derived from the frequencies’ calculation.

AMBER’s tLEAP module was used to generate an orthorhombic box of pre-equilibrated TIP3P [31] water molecules, including 9 Na^+^ ions, to neutralise the protein’s charge. Thus, a resulting box of 78.7 × 116.5 × 94.7 Å^3^ containing almost 72,000 atoms, around 9700 of them coming from the protein, was obtained. Bonds between substrate’s epoxide and WAT1′s H and substrate’s epoxide and H of TYR383 were manually added by adding bond force constants of 25 kcal/mol·Å^2^ in order to describe the detected H-bonds between these atoms.

MD calculations were performed in GPUs using the PMEMD module [57,58] in its CUDA version from the AMBER18 package.

Three initial MM minimisations were performed, combining the steepest descent method and the conjugate gradient method for 5000 steps each. In the first one, the protein, cofactors and substrate were kept frozen by applying a restraint of 150.0 kcal/mol·Å^2^. In the second one, only the backbone was restrained, while in the third minimisation, all the system was set free to move. A cut-off of 9 Å was applied in the three minimisations. Then, the system was heated from 0 to 300 K in steps of 30 K for 200 ps. SHAKE algorithm [59] was deactivated for the H of WAT and of TYR383, which are bonded to the epoxide. A restraint of 5 kcal/mol·Å^2^ was applied to the backbone, and a cut-off of 9 Å was used.

Once heated, an NPT step of 5 ns was performed at 1 bar and at 300 K with a restraint of 5 kcal/mol·Å^2^ applied to the backbone, a cut-off of 9 Å and with the SHAKE algorithm deactivated for the hydrogens bonded to the substrate’s epoxide. A density of 1.0025 g/mL was achieved, and so the system was equilibrated. The temperature was controlled by Langevin dynamics [60] while the pressure was adjusted by the Berendsen barostat [61].

Finally, an NVT equilibration step was performed before the NVT production step. Both share the same configuration: 300 K, no restraints and no SHAKE algorithm for hydrogens bonded to substrate’s epoxide. A total of 10 ns of equilibration were calculated followed by 150 ns of production, from which the last 100 ns have been selected for further analysis.

## 4. Conclusions

LTB_4_ is a very potent lipid inflammatory mediator involved in acute and chronic inflammatory diseases. The chirality of the 12*R*-hydroxyl group and the 6*Z*,8*E*,10*E* configuration of the conjugated triene moiety are key features for its bioactivity. LTB_4_ is obtained when LTA_4_ is hydrolysed by the enzyme LTA_4_H. In this paper, our Molecular Dynamics simulations and Quantum Mechanics/Molecular Mechanics calculations allowed us to theoretically capture LTA_4_H in action, revealing the molecular details of the complete step-by-step mechanism of that enzyme reaction, in good agreement with the available experimental results.

The first step of the reaction consists of the opening of the epoxide of LTA_4_. In a second, well-separated step, a water molecule is added to C_12_ of LTA_4_, while one of its hydrogen atoms is fully transferred to ASP375. The set of these two orthogonal steps constitutes a rather unusual 1,7-nucleophilic substitution through a clear S_N_1 mechanism.

In LTA_4_, the bond C_6_-C_7_ has a configuration rather similar to pro-*cis*, although intermediate between the pro-*cis* and the pro-*trans* ones. As a result of the epoxide opening, the bond C_6_-C_7_ evolves either to a *cis* (*Z*) or to a *trans* (*E*) configuration. The triene moiety must twist to allow the bond C_6_-C_7_ to adopt its *cis* (*Z*) or *trans* (*E*) configuration, in such a way that it exposes the *R* face of C_12_ (by the side opposite to the Zn atom) or the *S* face of C_12_ (by the same side as the Zn atom), respectively, to the water addition. Only the *cis* (*Z*) configuration will lead to LTB_4_ (5*S*,12*R*-dihydroxy-6*Z*,8*E*,10*E*,14*Z*-eicosatetraenoic acid), whereas the *trans* (*E*) configuration would eventually form its stereoisomer 12*S*-6-*trans*-LTB_4_ (5*S*,12*S*-dihydroxy-6*E*,8*E*,10*E*,14*Z*-eicosatetraenoic acid). Thus, the two main stereochemical features that are required for the bioactivity of LTB_4_ appear to be closely linked.

In the gas phase, 12*S*-6-*trans*-LTB_4_ is more stable than LTB_4_. Inside LTA_4_H, the epoxide opening (first step) to give the *trans* (*E*) configuration of the bond C_6_-C_7_ turns out to be more favourable than the opening to the *cis* (*Z*) configuration, both kinetically and thermodynamically. However, from here on, the *cis* energy profile becomes clearly below the *trans* energy profile. This can be explained through noncovalent π-π stacking interactions between the triene moiety and TYR267 and, especially, between the triene moiety and TYR378. Both tyrosines wrap the triene system along the whole reaction.

Finally, in the third step, the final transfer of a proton from a water molecule to the oxygen atom bonded to C_6_ (the former oxygen atom of the epoxide) takes place, thus forming LTB_4_. This proton transfer does not occur in the first step of the reaction to trigger an S_N_1 acid-induced opening of the epoxide ring

This is an excellent example of how the role of an enzyme is not only to accelerate the reaction rate, but to govern the stereochemistry of the product, making it possible for the bioactive product (LTB_4_ in this case) to be formed.

## Figures and Tables

**Table 1:** Most relevant distances (in Å) and dihedral angle (in degrees) defining the *E*/*Z* character of the C_6_-C_7_ bond for the stationary structures of the complete mechanism of conversion of LTA_4_ to LTB_4_ (pro-*cis* reaction path) catalysed by LTA_4_H.

Pro-*cis* Reaction Path	R	TS1	INT1	TS2	INT2	TS3	P
Zn-O_epox_	3.39	2.94	1.99	1.97	1.95	2.45	2.96
O_epox_-C_5_	1.45	1.41	1.39	1.39	1.39	1.41	1.43
O_epox_-C_6_	1.47	1.92	2.40	2.41	2.42	2.37	2.39
H(WAT1)-O_epox_	1.75	1.65	2.42	2.46	2.49	1.39	1.02
O(WAT1)-H(WAT1)	0.99	1.00	0.97	0.97	0.98	1.09	1.59
O(WAT3)-C_12_	3.84	3.69	3.69	1.82	1.53	1.53	1.53
H(WAT3)-O(ASP375)	1.71	1.69	1.67	1.36	1.03	1.03	1.03
O(WAT3)-H(WAT3)	0.99	0.99	0.99	1.11	1.53	1.53	1.53
H_6_-C_6_-C_7_-H_7_	−65.6	−15.9	−7.1	1.5	1.5	−0.5	−0.7

**Table 2:** Most relevant distances (in Å) and dihedral angle (in degrees) defining the *E*/*Z* character of the C_6_-C_7_ bond for the stationary structures of the pro-*trans* reaction path catalysed by LTA_4_H.

Pro-*trans* Reaction Path	R	TS1	INT1	TS2	INT2	TS3	P
Zn-O_epox_	3.39	2.64	1.98	1.93	1.92	3.26	2.96
O_epox_-C_5_	1.45	1.40	1.39	1.39	1.39	1.44	1.43
O_epox_-C_6_	1.47	2.04	2.20	2.30	2.32	2.39	2.34
H(WAT1)-O_epox_	1.75	1.66	3.00	3.12	3.12	0.97	1.02
O(WAT1)-H(WAT1)	0.99	1.00	0.98	0.98	0.98	2.39	1.59
O(WAT3)-C_12_	3.84	3.68	3.66	1.79	1.51	1.50	1.50
H(WAT3)-O(ASP375)	1.71	1.66	1.66	1.32	1.01	1.01	1.01
O(WAT3)-H(WAT3)	0.99	1.00	1.00	1.12	1.60	1.64	1.62
H_6_-C_6_-C_7_-H_7_	294.4	196.9	183.7	178.1	177.5	180.7	181.5

## Data Availability

The data presented in this study are contained within the article.

## Supplementary Materials

The following supporting information can be downloaded at: https://www.mdpi.com/article/10.3390/ijms23063140/s1.
