# Supplementary material for: Theoretical Characterization of the Step-by-Step Mechanism of Conversion of Leukotriene A4 to Leukotriene B4 Catalysed by the Enzyme Leukotriene A4 Hydrolase
Source: Int J Mol Sci. 2022 Mar 15;23(6):3140. doi: 10.3390/ijms23063140 (PMC8954237; doi:10.3390/ijms23063140)
Supplement: Supplementary file 1 [file ijms-23-03140-s001.zip › ijms-1622937-supplementary.pdf]

Supplementary Materials

Theoretical Characterization of the Step-By-Step Mechanism of Conversion of Leukotriene A4 to Leukotriene B4 Catalysed by the Enzyme Leukotriene A4 Hydrolase

Miquel Canyelles-Niño[1,2], Àngels González-Lafont [1,3] and José M. Lluch [1,3,*]

[1] Departament de Química, Universitat Autònoma de Barcelona, 08193 Bellaterra, Barcelona, Spain; Miquel.Canyelles@uab.cat (M.C.-N.); Angels.Gonzalez@uab.cat (À.G.-L.); JoseMaria.Lluch@uab.cat (J.M.L.).
[2] Biochemize SL, Zamora 45, 08005 Barcelona, Spain.
[3] Institut de Biotecnologia i de Biomedicina (IBB), Universitat Autònoma de Barcelona, 08193 Bellaterra, Barcelona, Spain.

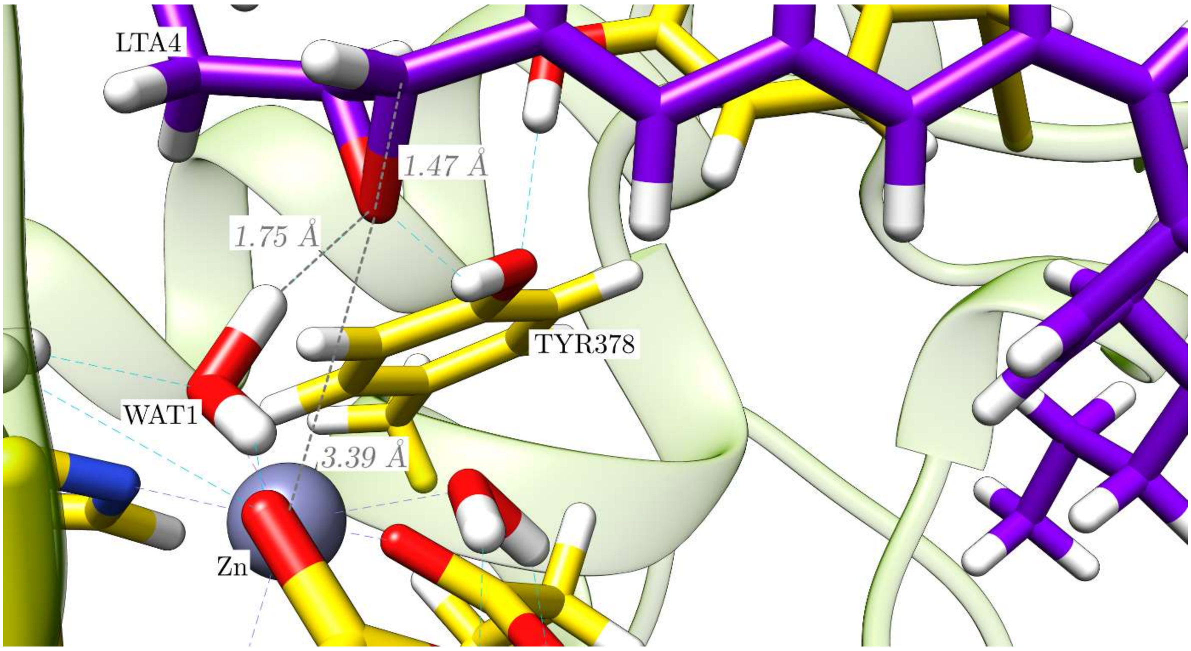

R

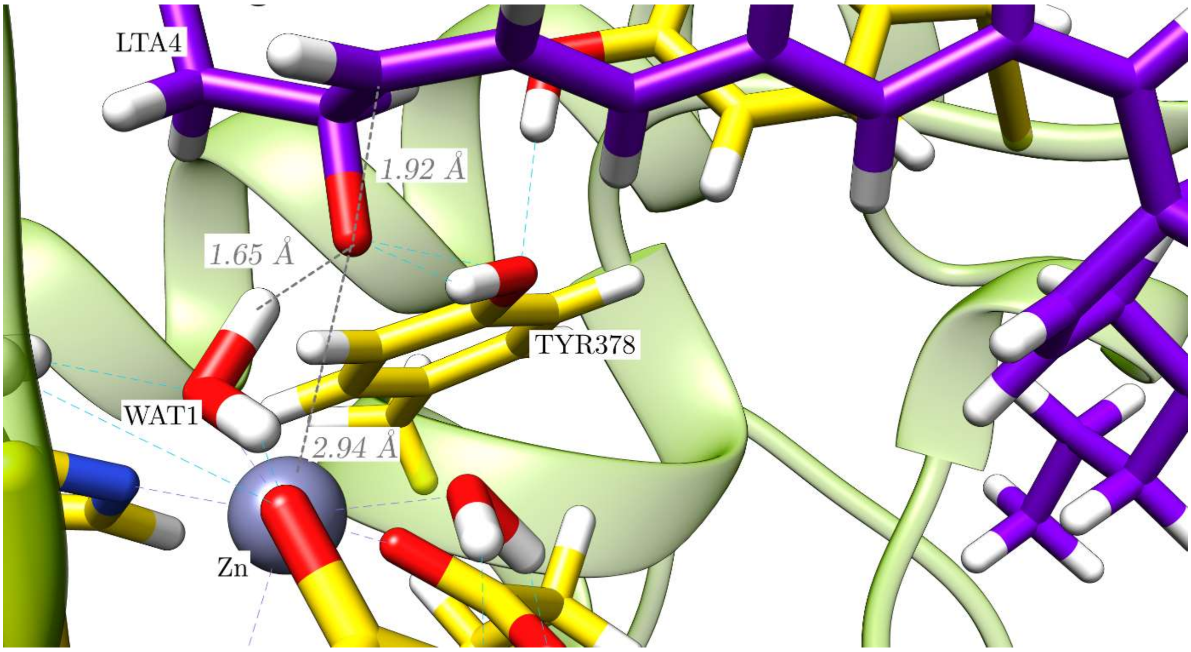

TS1cis

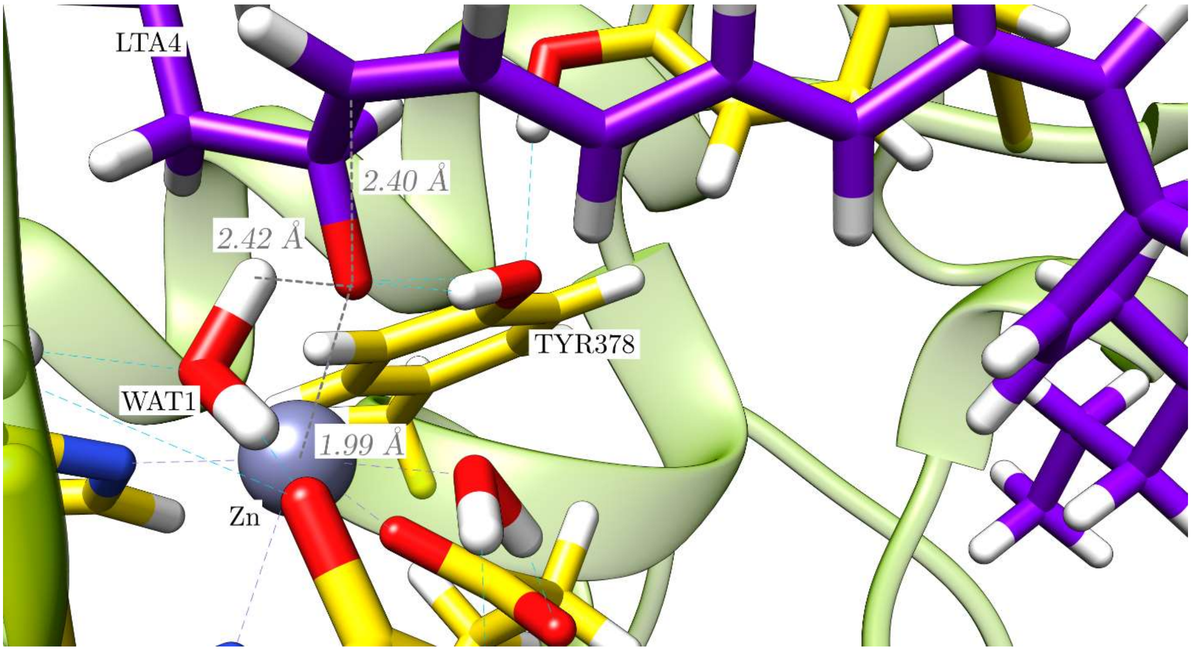

INT1cis

Figure S1. Representation of the stationary structures R, TS1cis and INT1cis focused on the region corresponding to the epoxide ring opening.

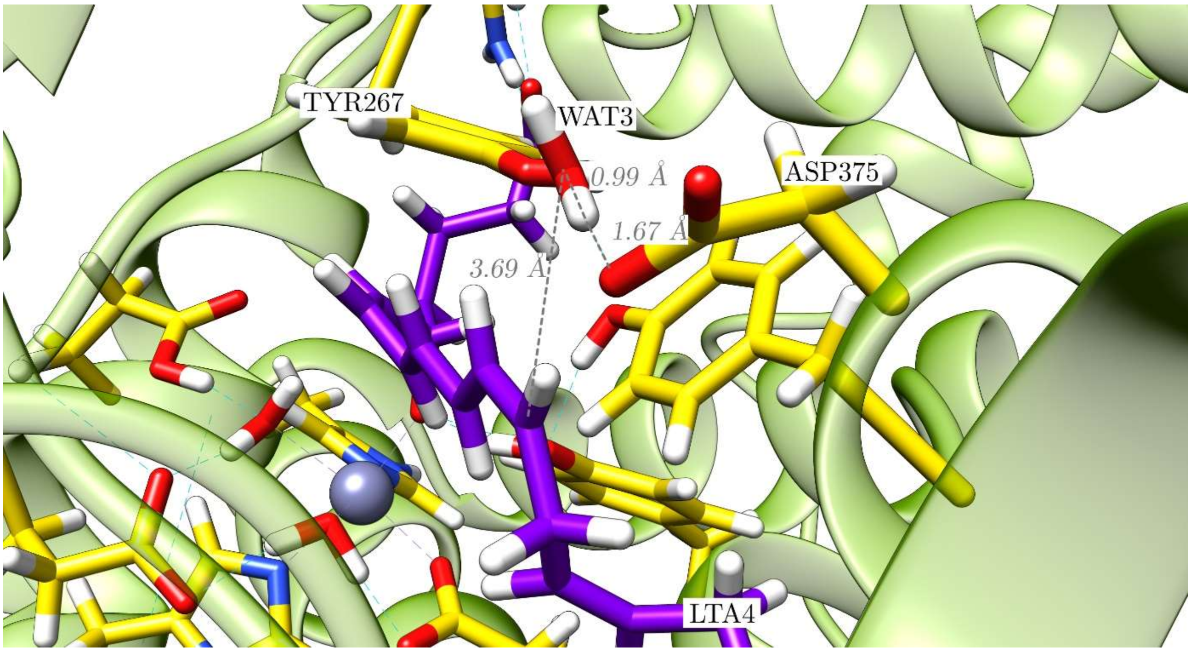

INT1cis

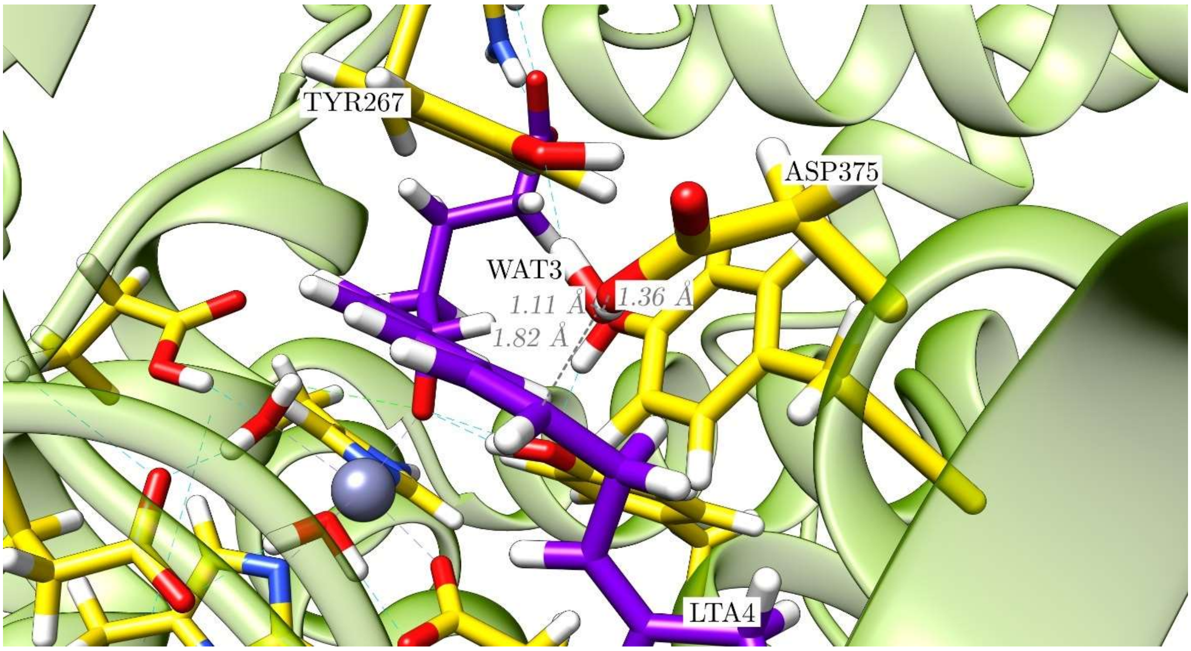

TS2cis

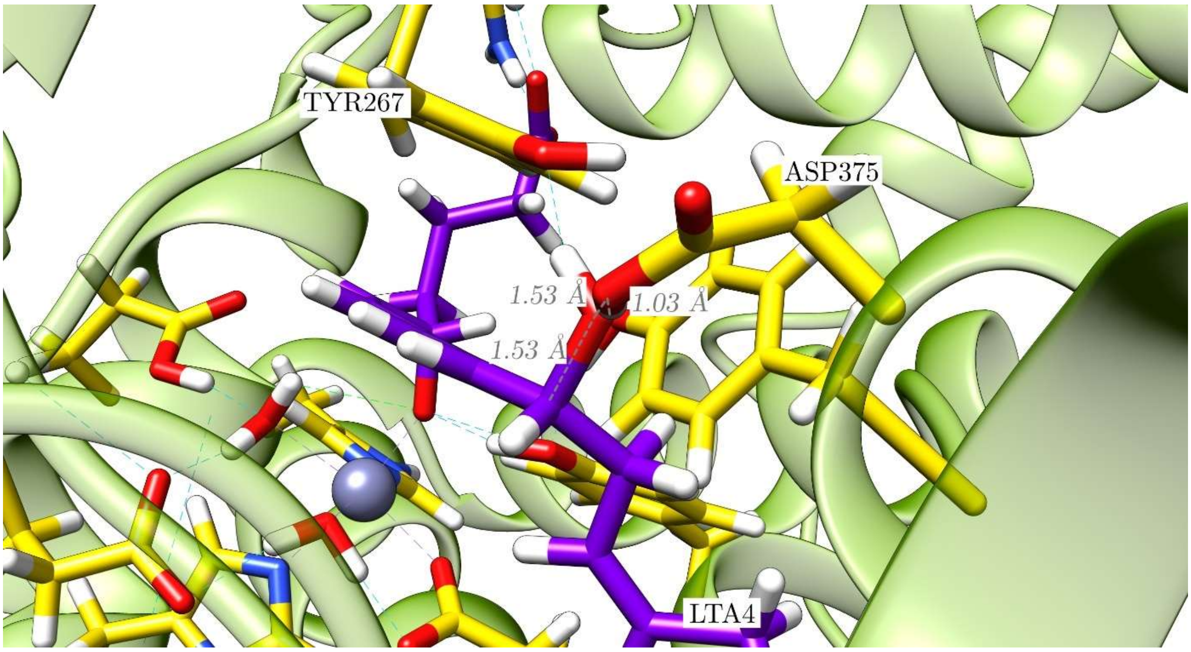

INT2cis

Figure S2. Representation of the stationary structures INT1cis, TS2cis and INT2cis focused on the region corresponding to the addition of WAT3 to C_{12} of the substrate.

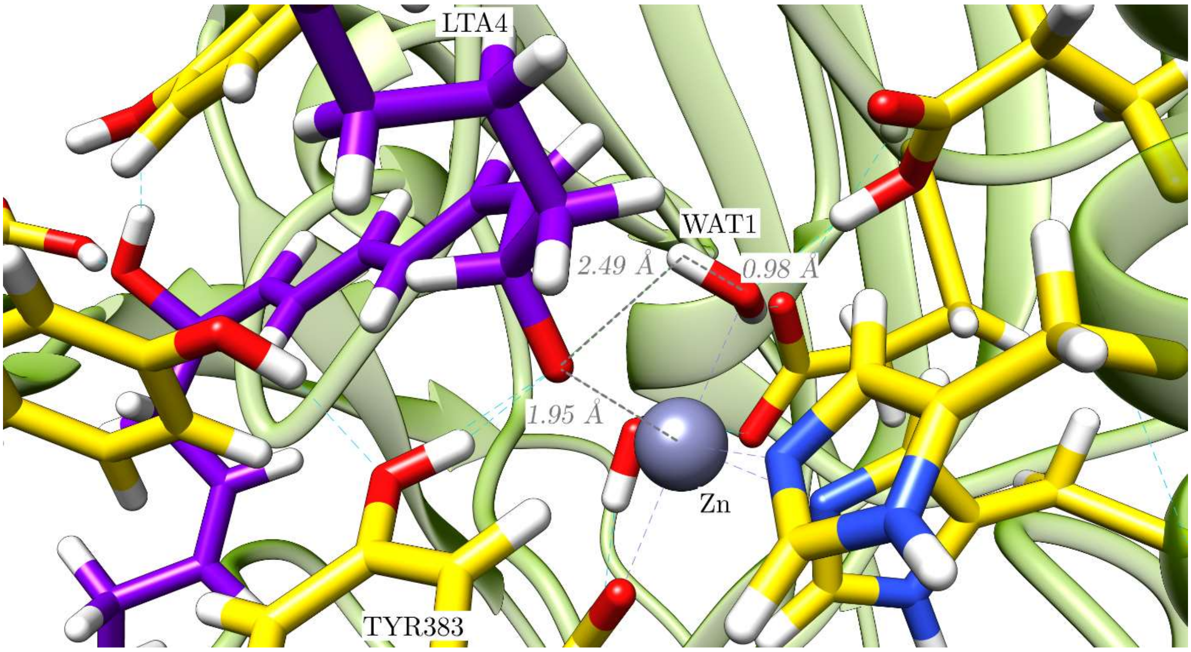

INT2cis

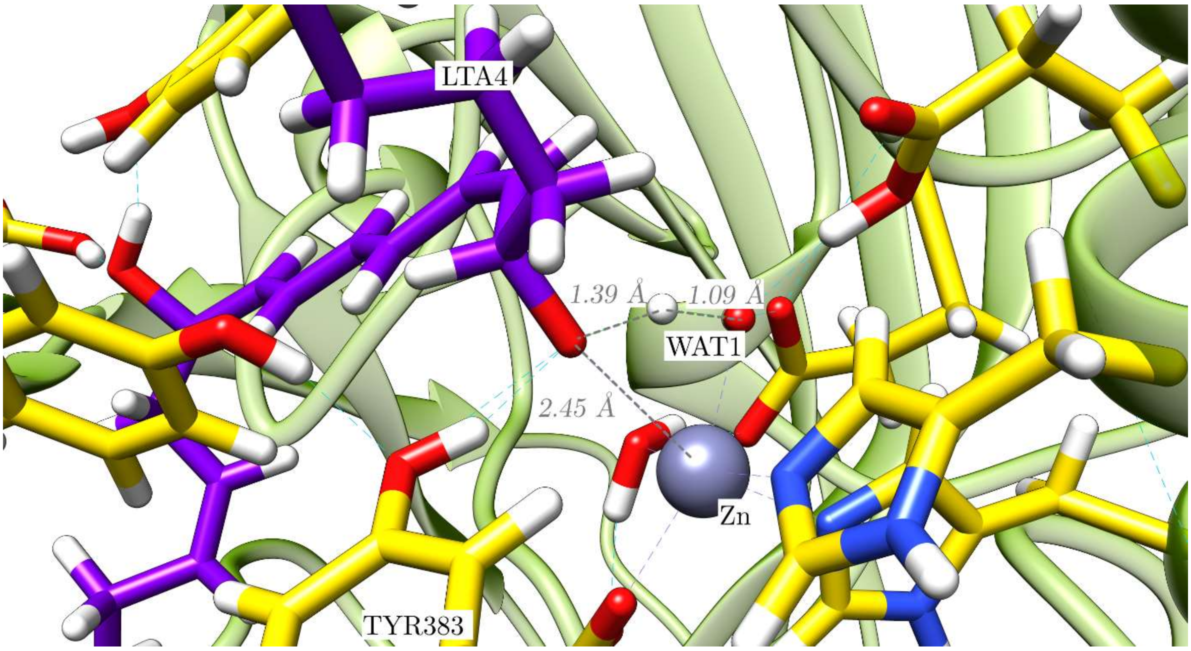

TS3cis

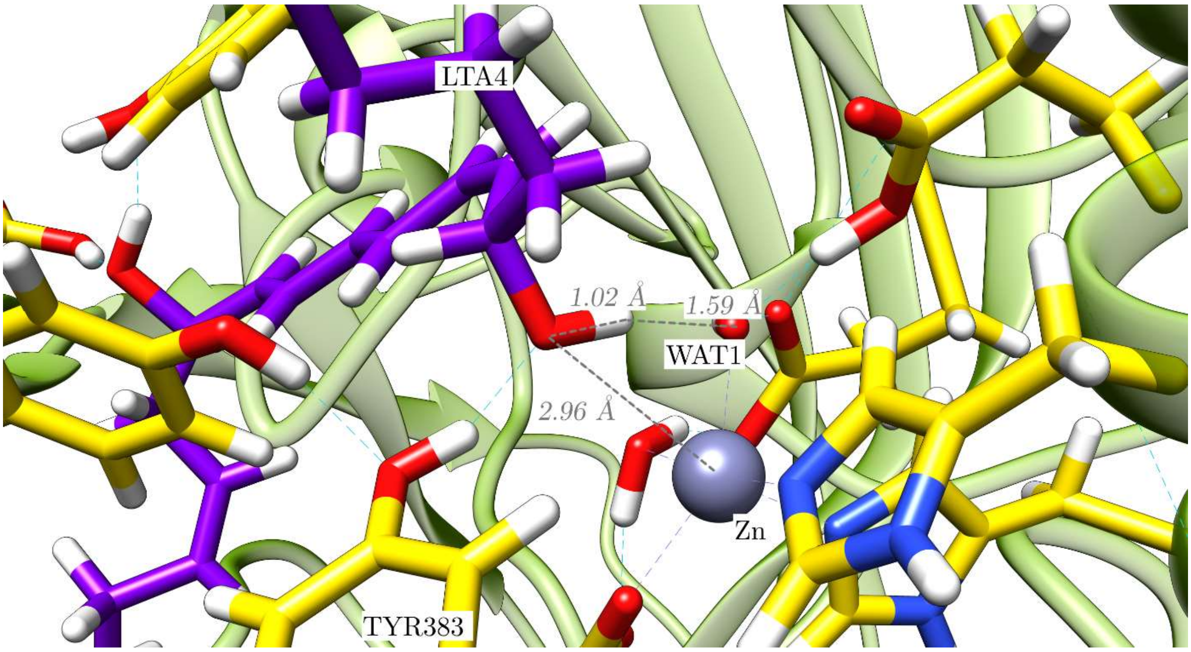

Pcis

Figure S3. Representation of the stationary structures INT2cis, TS3cis and Pcis focused on the region corresponding to the final protonation of the oxygen atom bonded to C_6 (the former oxygen atom of the epoxide).

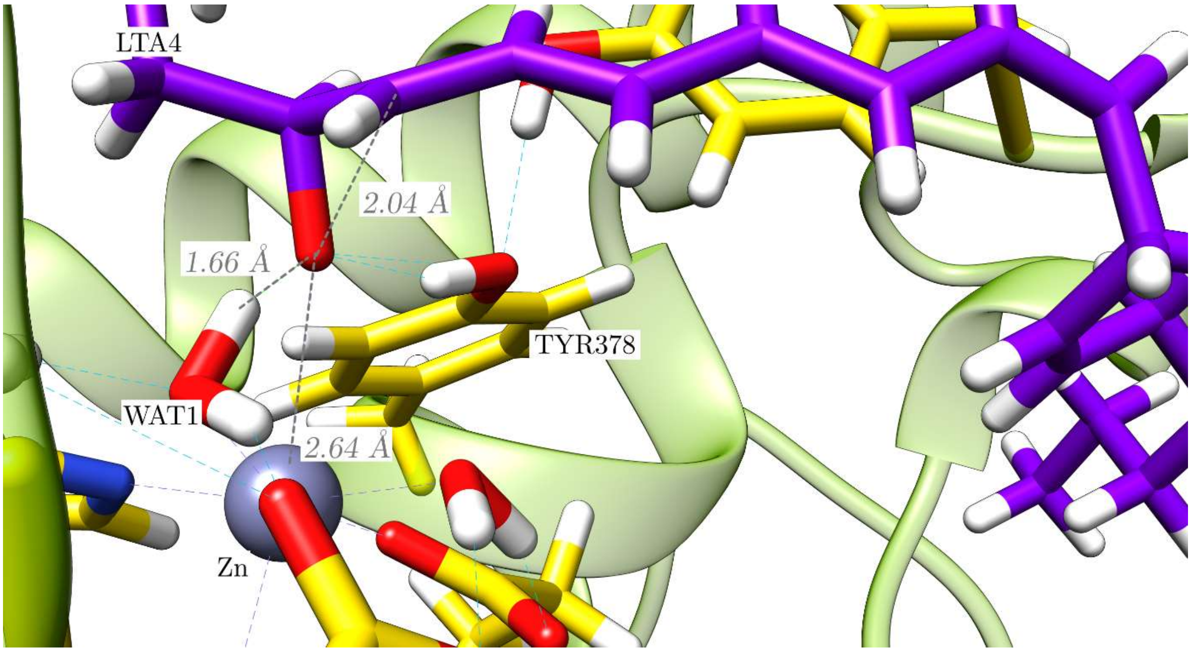

TS1trans

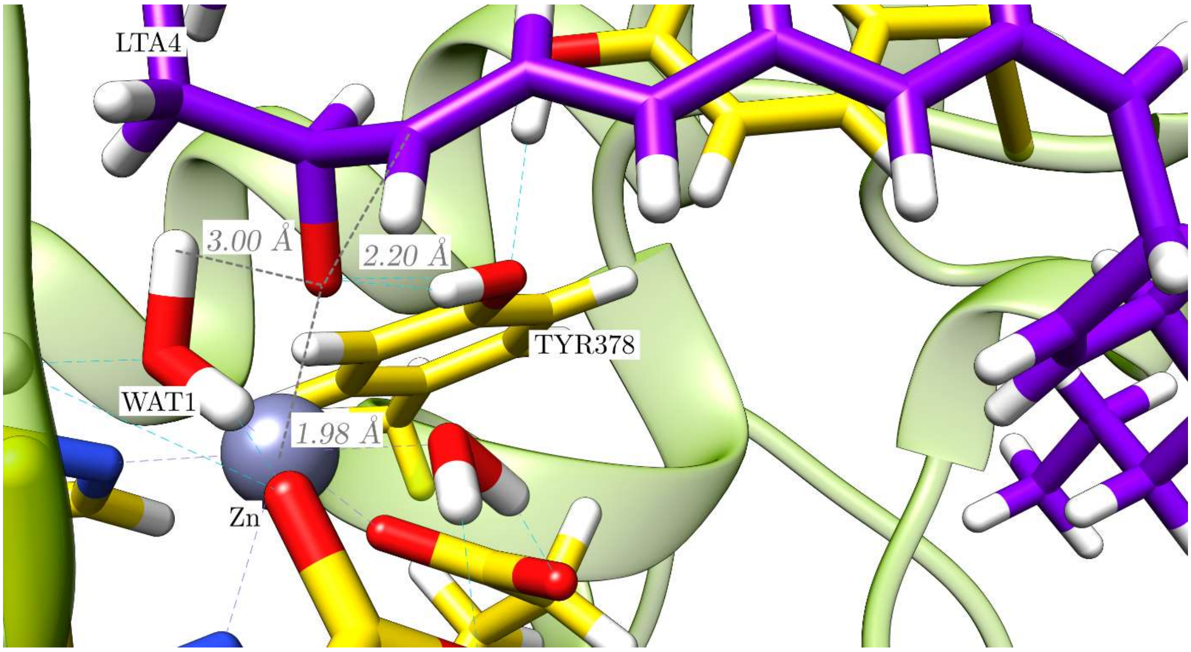

INT1trans

Figure S4. Representation of the stationary structures TS1trans and INT1trans focused on the region corresponding to the epoxide ring opening.

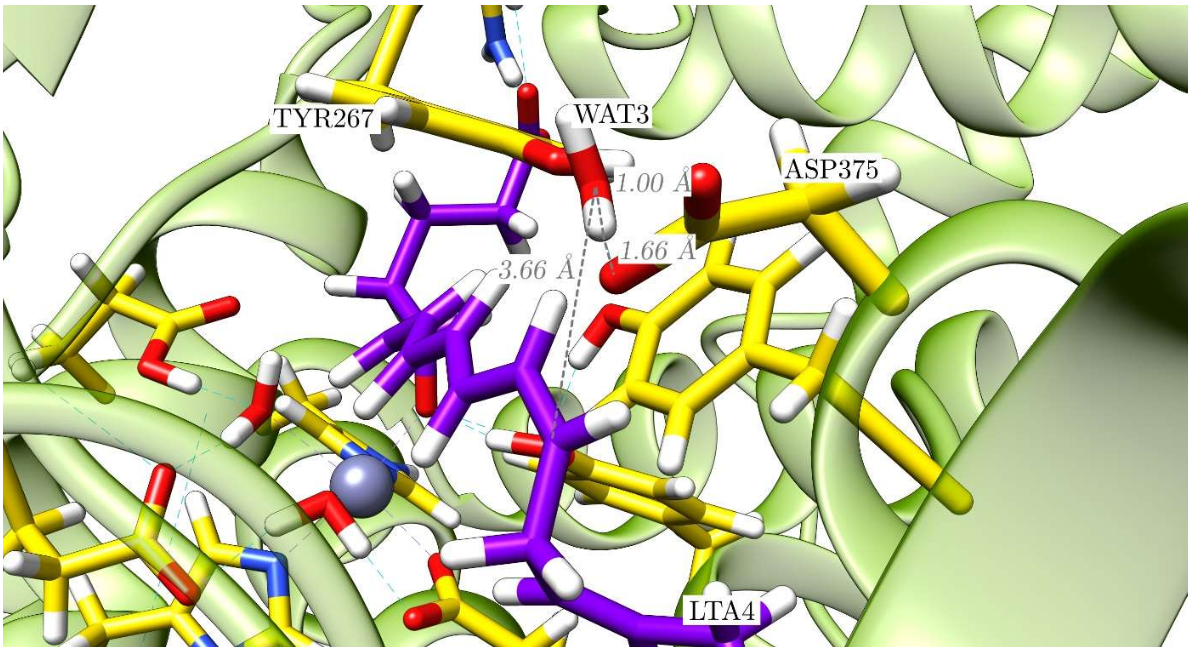

INT1trans

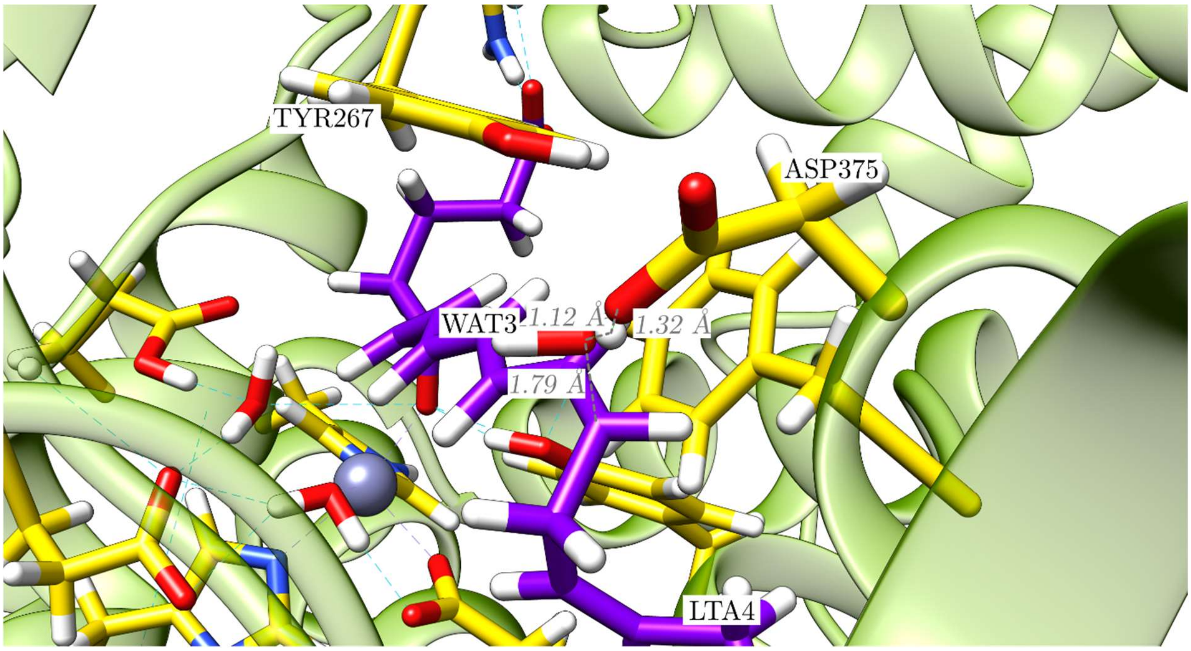

TS2trans

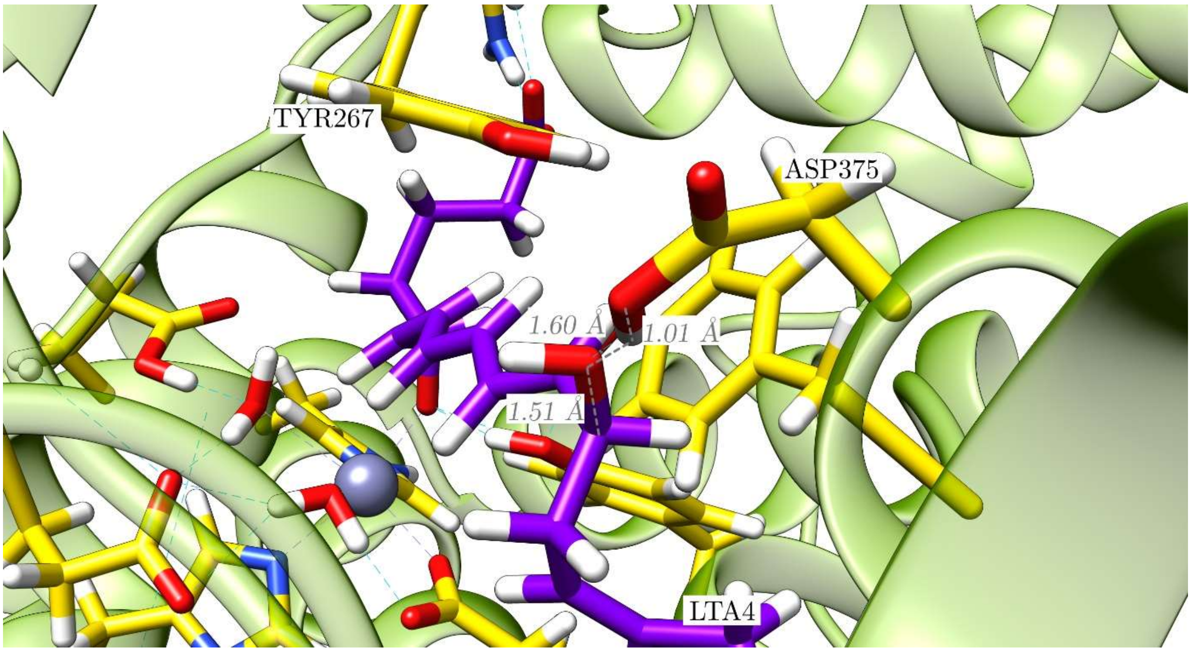

INT2trans

Figure S5. Representation of the stationary structures INT1trans, TS2trans and INT2trans focused on the region corresponding to the addition of WAT3 to C_{12} of the substrate.

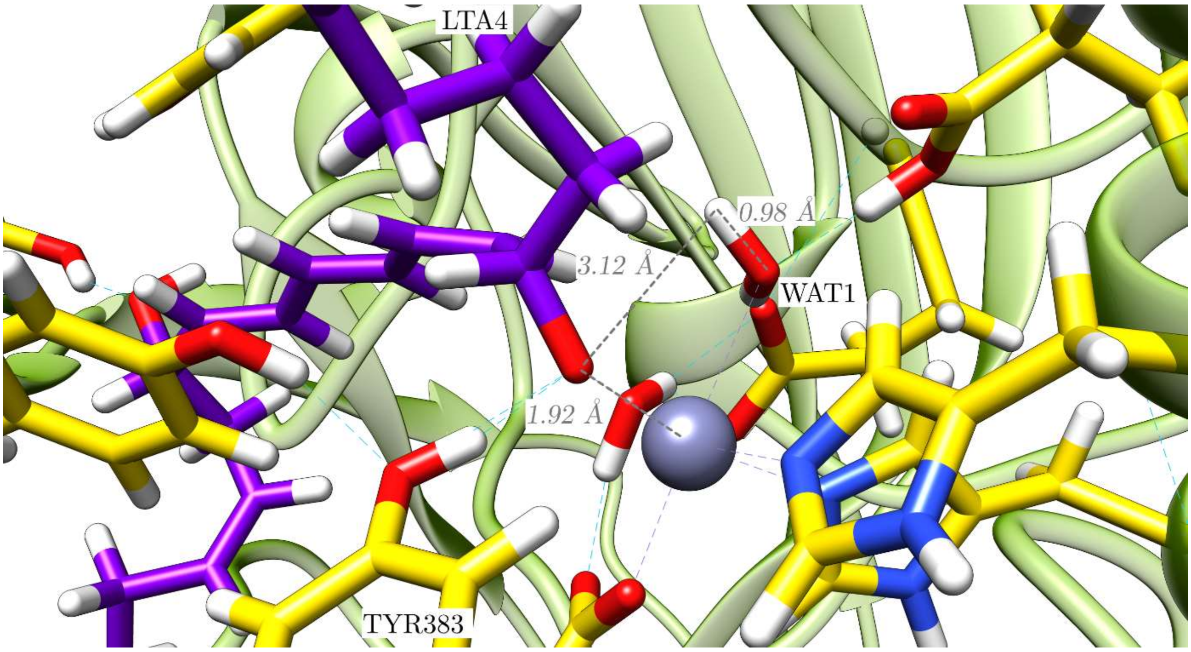

INT2trans

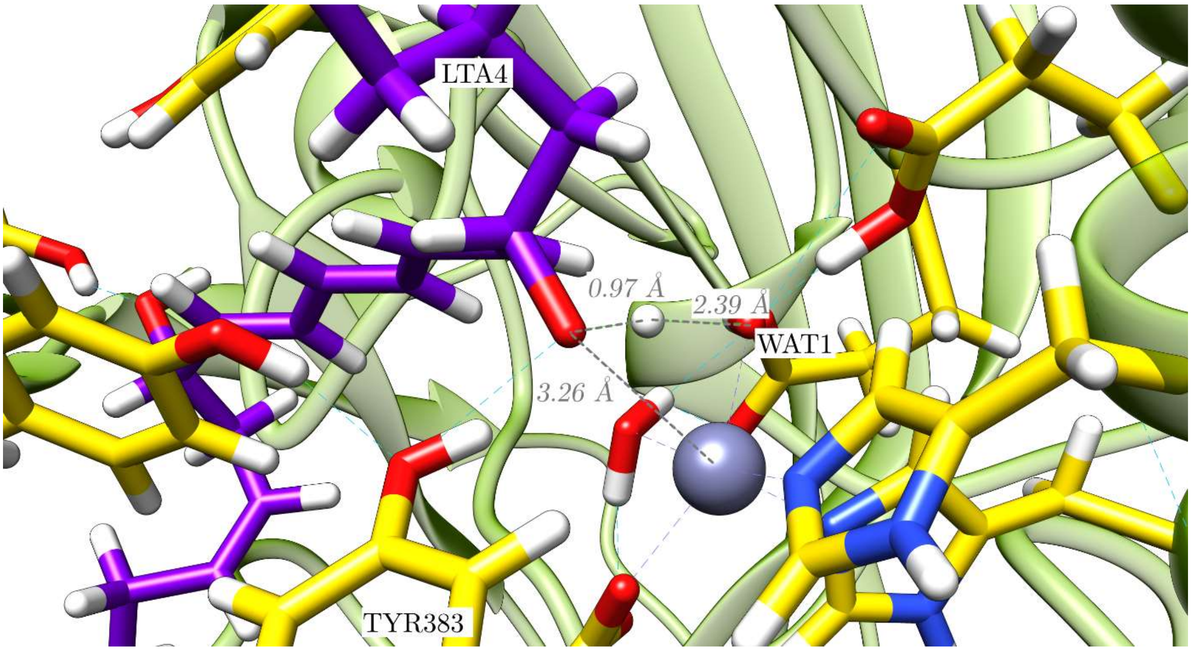

TS3trans

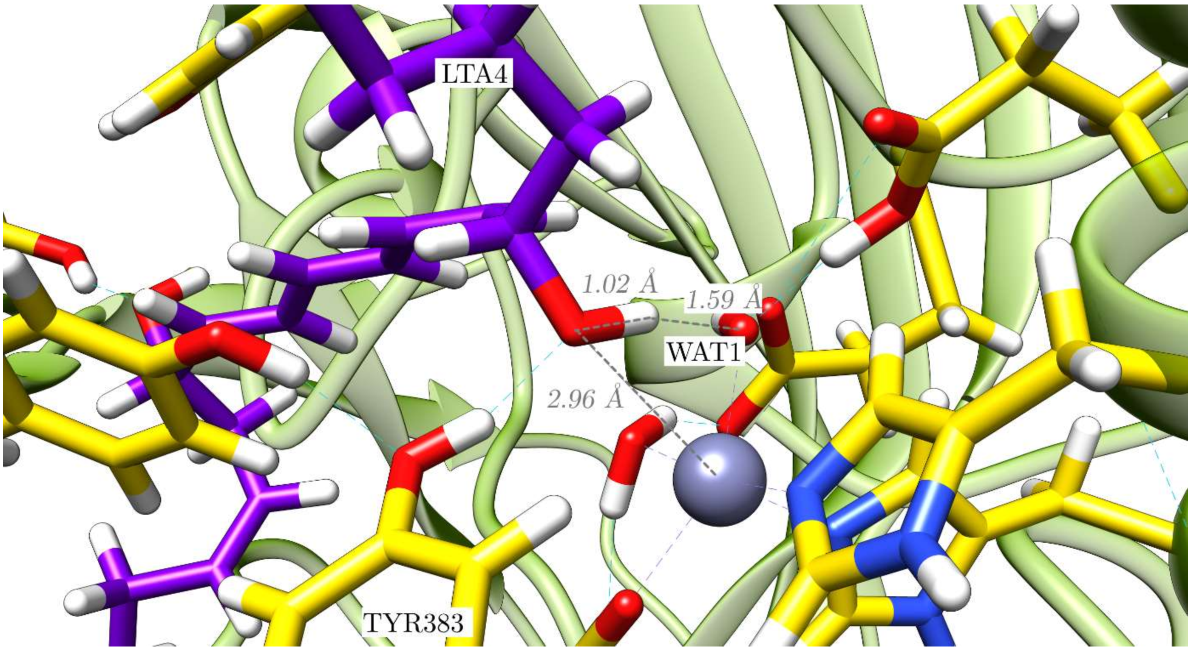

Ptrans

Figure S6. Representation of the stationary structures INT2trans, TS3trans and Ptrans focused on the region corresponding to the final protonation of the oxygen atom bonded to C_6 (the former oxygen atom of the epoxide).
